# Correction: Consumer perceptions of antimicrobial use in animal husbandry: A scoping review

**DOI:** 10.1371/journal.pone.0270442

**Published:** 2022-06-21

**Authors:** Jaime R. Barrett, Gabriel K. Innes, Kelly A. Johnson, Guillaume Lhermie, Renata Ivanek, Amelia Greiner Safi, David Lansing

There is an error in affiliation for author Amelia Greiner Safi. The correct affiliations are: **1** Department of Public and Ecosystem Health, College of Veterinary Medicine, Cornell University, Ithaca, NY, United States of America, **2** Department of Communication, College of Agriculture and Life Sciences Cornell University, Ithaca, NY, United States of America.
