# Supplementary material for: Consumer perceptions of antimicrobial use in animal husbandry: A scoping review
Source: PLoS One. 2021 Dec 8;16(12):e0261010. doi: 10.1371/journal.pone.0261010 (PMC8654221; doi:10.1371/journal.pone.0261010)
Supplement: S1 File — This contains citations for all 124 retained texts used for analysis. (DOCX) [file pone.0261010.s001.docx]

**S1 File: Bibliography of final texts**

Abrams, K. M., Meyers, C. A., & Irani, T. A. (2010). Naturally confused: Consumers’ perceptions of all-natural and organic pork products. *Agriculture and Human Values*, *27*(3), 365–374. ABI/INFORM Collection. <https://doi.org/10.1007/s10460-009-9234-5>

Adamski, M., Kuzniacka, J., & Milczewska, N. (2017). Preferences of consumers for choosing poultry meat. *Polish Journal of Natural Sciences*, *32*(2), 261–271.

Anon. (1993). Performance enhancers—Their impact on farmers and consumers. *Misset World Poultry*, *9*(11).

Ardeshiri, A., Sampson, S., & Swait, J. (2019). Seasonality effects on consumers’ preferences over quality attributes of different beef products. *Meat Science*, *157*, 107868. <https://doi.org/10.1016/j.meatsci.2019.06.004>

Argemi-Armengol, I., Villalba, D., Ripoll, G., Teixeira, A., & Alvarez-Rodriguez, J. (2019). Credence cues of pork are more important than consumers’ culinary skills to boost their purchasing intention. *Meat Science*, *154*, 11–21. <https://doi.org/10.1016/j.meatsci.2019.04.001>

Barlow, J. (2011). Antimicrobial Resistance and the Use of Antibiotics in the Dairy Industry: Facing Consumer Perceptions and Producer Realities. In L. Doepel (Ed.), *Advances in Dairy Technology, Vol 23* (Vol. 23, pp. 47–58).

Becker, T., Benner, E., & Glitsch, K. (2000). Consumer perception of fresh meat quality in Germany. *British Food Journal*, *102*(3), 246–266. <https://doi.org/10.1108/00070700010324763>

Begemann, S., Perkins, E., Hoyweghen, I. van, Christley, R., & Watkins, F. (2018). How political cultures produce different antibiotic policies in agriculture: A historical comparative case study between the United Kingdom and Sweden. *Sociologia Ruralis*, *58*(4), 765–785. <https://doi.org/10.1111/soru.12206>

Belcher, K. W., Germann, A. E., & Schmutz, J. K. (2007). Beef with environmental and quality attributes: Preferences of environmental group and general population consumers in Saskatchewan, Canada. *Agriculture and Human Values*, *24*(3), 333–342. ABI/INFORM Collection. <https://doi.org/10.1007/s10460-007-9069-x>

Bergstra, T., Hogeveen, H., Kuiper, W. E., Lansink, A. G. J. M. O., & Stassen, E. N. (2017). Attitudes of Dutch Citizens toward Sow Husbandry with Regard to Animals, Humans, and the Environment. *Anthrozoos*, *30*(2), 195–211. <https://doi.org/10.1080/08927936.2017.1310985>

Bernard, J.C., Pan, X., & Pesek, J. D. Jr. (2007). Consumer likelihood to purchase chickens with novel production attributes. *Journal of Agricultural and Applied Economics*, *39*(3), 581–596. agr.

Bernard, John C., & Bernard, D. J. (2009). What Is It About Organic Milk? An Experimental Analysis [electronic resource]. *American Journal of Agricultural Economics*, *91*(3), 826–836. agr.

Bernard, John C., Xiqian Pan, & Sirolli, R. (2005). Consumer Attitudes Toward Genetic Modification and Other Possible Production Attributes for Chicken. *Journal of Food Distribution Research*, *36*(2), 1–11. bth.

Bir, C. L., Widmar, N. J. O., Davis, M. K., Erasmus, M. A., & Zuelly, S. (2020). Willingness to pay for whole turkey attributes during Thanksgiving holiday shopping in the United States. *Poultry science*, *99*(5), 2798-2810. <https://dx.doi.org/10.1016/j.psj.2019.12.047>

Bir, C., Widmar, N. O., Thompson, N. M., Townsend, J., & Wolf, C. A. (2020). US respondents' willingness to pay for Cheddar cheese from dairy cattle with different pasture access, antibiotic use, and dehorning practices. *Journal of dairy science*, *103*(4), 3234-3249. <https://dx.doi.org/10.3168/jds.2019-17031>

Blair, R. (2011). *Organic production and food quality. A down to earth analysis.*

Bourne, J. E., Ginis, K. A. M., Buchholz, A. C., & Jung, M. E. (2018). Strategies for public health initiatives targeting dairy consumption in young children: A qualitative formative investigation of parent perceptions. *Maternal and Child Nutrition*, *14*(Suppl. S2). <https://doi.org/10.1111/mcn.12587>

Bowman, M., Marshall, K. K., Kuchler, F., & Lynch, L. (2016). Raised Without Antibiotics: Lessons from Voluntary Labeling of Antibiotic Use Practices in The Broiler Industry. *American Journal of Agricultural Economics*, *98*(2), 622. ABI/INFORM Collection.

Brewer, M. S., & Rojas, M. (2008). Consumer attitudes toward issues in food safety. *Journal of Food Safety*, *28*(1), 1–22. <https://doi.org/10.1111/j.1745-4565.2007.00091.x>

Bryant, C., van Nek, L., & Rolland, N. (2020). European markets for cultured meat: A comparison of Germany and France. *Foods*, *9*(9), 1152. <https://dx.doi.org/10.3390/foods9091152>

Bulut, E., Stout, A., Wemette, M., Llanos‐Soto, S., Schell, R. C., Greiner Safi, A., ... & Ivanek, R. (2021). How does public perception of antibiotic use on dairy farms contribute to self‐reported purchasing of organic?. *Journal of Food Science*. <https://dx.doi.org/10.1111/1750-3841.15720>

Busch, G., Kassas, B., Palma, M. A., & Risius, A. (2020). Perceptions of antibiotic use in livestock farming in Germany, Italy and the United States. *Livestock Science*, *241*, 104251. <https://dx.doi.org/10.1016/j.livsci.2020.104251>

Canada, S. (2018, May 10). *What we heard—Full report*. Aem. <https://www.canada.ca/en/campaign/food-policy/consulting-with-canadians/fullreport.html>

Cargill Feed for Thought Survey: US, Brazilian Consumers More Likely to Buy Beef Raised Without Antibiotics. (2016). *Food Manufacturing*. ABI/INFORM Collection. <https://search.proquest.com/docview/1813584948?accountid=10267>

Chicken consumption survey reveals purchase influences: The National Chicken Council’s survey of chicken usage reveals shifts in consumption behavior and consumer attitudes about purchase criteria and product claims. (2017, November). *Poultry USA*. ABI/INFORM Collection. <https://search.proquest.com/docview/2112928272?accountid=10267>

Christoph-Schulz, I., Salamon, P., & Weible, D. (2015). What is the benefit of organically-reared dairy cattle? Societal perception towards conventional and organic dairy farming. *International Journal on Food System Dynamics*, *6*(3), 139–146.

Christoph-Schulz, I., & Rovers, A. K. (2020). German Citizens’ Perception of Fattening Pig Husbandry—Evidence from a Mixed Methods Approach. *Agriculture*, *10*(8), 342. <https://dx.doi.org/10.3390/agriculture10080342>

Claret, A., Guerrero, L., Gines, R., Grau, A., Hernandez, M. D., Aguirre, E., Peleteiro, J. B., Fernandez-Pato, C., & Rodriguez-Rodriguez, C. (2014). Consumer beliefs regarding farmed versus wild fish. *Appetite*, *79*, 25–31. <https://doi.org/10.1016/j.appet.2014.03.031>

Clark, B. (2018). *Production diseases and farm animal welfare: What do the public think?* (2176266497) [Ph.D., University of Newcastle Upon Tyne (United Kingdom)]. ProQuest Dissertations & Theses Global: Business; ProQuest Dissertations & Theses Global: Health & Medicine; ProQuest Dissertations & Theses Global: History; ProQuest Dissertations & Theses Global: Literature & Language; ProQuest Dissertations & Theses Global: Science & Technology; ProQuest Dissertations & Theses Global: Social Sciences; ProQuest Dissertations & Theses Global: The Arts. <https://search.proquest.com/docview/2176266497?accountid=10267>

Clark, B., Panzone, L. A., Stewart, G. B., Kyriazakis, I., Niemi, J. K., Latvala, T., Tranter, R., Jones, P., & Frewer, L. J. (2019). Consumer attitudes towards production diseases in intensive production systems. *PLoS One*, *14*(1), e0210432. <https://doi.org/10.1371/journal.pone.0210432>

Conner, D. S., Campbell-Arvai, V., & Hamm, M. W. (2008). Consumer preferences for pasture-raised animal products: Results from Michigan. *Journal of Food Distribution Research*, *39*(2), 12–25.

Consumer Reports Poll: Majority Of Americans Want Meat Raised Without Antibiotics Sold At Local Supermarkets. (2012, June 20). *PR Newswire*. ABI/INFORM Collection. <https://search.proquest.com/docview/1021115208?accountid=10267>

CONWAY, A. (2017). What poultry purchases do consumers really make? *WATT PoultryUSA*, *18*(12), 32–36. bth.

Coulter, R. L., Coulter, M. K., & Murrow, J. L. (2002). The Food Animal Supply and Its Related Concerns: Understanding the American Perspective. *Journal of Food Products Marketing*, *8*(2), 33. bth.

Cowan, C. (1998). Irish and European consumer views on food safety. *Journal of Food Safety*, *18*(4), 275–295. <https://doi.org/10.1111/j.1745-4565.1998.tb00221.x>

Cowan, C., Mannion, M., Langan, J., & Keane, J. B. (1999). Consumer perceptions of meat quality. *Consumer Perceptions of Meat Quality.*

Dahlhausen, J. L., Rungie, C., & Roosen, J. (2018). Value of labeling credence attributes-common structures and individual preferences. *Agricultural Economics*, *49*(6), 741–751. <https://doi.org/10.1111/agec.12456>

Dennis, E. J., Ajewole, K., Bergtold, J. S., & Schroeder, T. C. (2020). Consumer Reactions to E. Coli and Antibiotic Residue Recalls: Utility Maximization vs. Regret Minimization. *Frontiers in veterinary science*, *7*, 611. <https://dx.doi.org/10.3389/fvets.2020.00611>

Denver, S., Jensen, J. D., & Christensen, T. (2021). Consumer preferences for reduced antibiotic use in Danish pig production. *Preventive Veterinary Medicine*, *189*, 105310. <https://dx.doi.org/10.1016/j.prevetmed.2021.105310>

Dohle, S., Campbell, V. E. A., & Arvai, J. L. (2013). Consumer-perceived risks and choices about pharmaceuticals in the environment: A  cross-sectional study. *Environmental Health : A Global Access Science Source*, *12*, 45. <https://doi.org/10.1186/1476-069X-12-45>

Dougan, N. Y. (2018). *Antibiotic Usage in Poultry and Eggs: Perception of Risk and Willingness to Pay for Alternatives* (2188069896) [Ph.D., George Mason University]. ProQuest Dissertations & Theses Global: Science & Technology; ProQuest Dissertations & Theses Global: Social Sciences. <https://search.proquest.com/docview/2188069896?accountid=10267>

Enough Movement Sponsored Survey Data Spotlights Rift Between Consumers’ Perceptions, Science about Food Choice. (2017). *Professional Services Close - Up*. ABI/INFORM Collection. <https://search.proquest.com/docview/1887319440?accountid=10267>

Etienne, J., Chirico, S., Gunabalasingham, T., Dautzenberg, S., & Gysen, S. (2017). EU insights—Perceptions on the human health impact of antimicrobial resistance (AMR) and antibiotics use in animals across the EU. *EFSA Supporting Publications*, *14*(3), EN-1183. <https://doi.org/10.2903/sp.efsa.2017.en-1183>

FAO. (n.d.). *Factors Driving Organic Agriculture Groth*.

Feucht, Y., & Zander, K. (2015). Of earth ponds, flow-through and closed recirculation systems—German consumers’ understanding of sustainable aquaculture and its communication. *Aquaculture*, *438*, 151–158. <https://doi.org/10.1016/j.aquaculture.2015.01.005>

Florkowski, W. J., Elnagheeb, A. H., & Huang, C. L. (1998). Risk perception and new food production technologies. *Applied Economics Letters*, *5*(2), 69–73. <https://doi.org/10.1080/758523506>

GAZDZIAK, S. (2016). Knowing what they DON’T want. *National Provisioner*, *230*(11), 20–26. bth.

Gifford, K., & Bernard, J. C. (2008). Factor and cluster analysis of willingness to pay for organic and non-GM food. *Journal of Food Distribution Research*, *39*(2), 26–39.

Glitsch, K. (2000). Consumer perceptions of fresh meat quality: Cross-national comparison. *British Food Journal*, *102*(3), 177–194. <https://doi.org/10.1108/00070700010332278>

Goddard, E., Muringai, V., & Boaitey, A. (2019). Moral foundations and credence attributes in livestock production: Canada. *The Journal of Consumer Marketing*, *36*(3), 418–428. ABI/INFORM Collection. <https://doi.org/10.1108/JCM-02-2018-2550>

Goddard, E., Muringai, V., & Robinson, A. (2017). Consumer Interest in a Natural Designation in Food Choice. *IDEAS Working Paper Series from RePEc*. ABI/INFORM Collection. <https://search.proquest.com/docview/2083125333?accountid=10267>

Govindasamy, R., DeCongelio, M., Italia, J., Barbour, B., & Anderson, K. (2001). Empirically Evaluating Consumer Characteristics and Satisfaction with Organic Products. *IDEAS Working Paper Series from RePEc*. ABI/INFORM Collection. <https://search.proquest.com/docview/1697527684?accountid=10267>

Grannis, J. L., & Thilmany, D. D. (1999). Targetable Market Segments For Natural Pork Products. *IDEAS Working Paper Series from RePEc*. ABI/INFORM Collection. <https://search.proquest.com/docview/1697460379?accountid=10267>

Halbrendt, C., Gempesaw, C. M., Bacon, R., & Sterling, L. (1991). Public perceptions of food safety in animal-food products. *Journal of Agribusiness*, *9*(1), 85–96.

Hans Stubbe Solgaard, & Yang, Y. (2011). Consumers’ perception of farmed fish and willingness to pay for fish welfare. *British Food Journal*, *113*(8), 997–1010. ABI/INFORM Collection. <https://doi.org/10.1108/00070701111153751>

Hwang, Y. J. (2006). *Three Essays on Economics and Risk Perception* (2047378364) [Ph.D., The Ohio State University]. ProQuest Dissertations & Theses Global: Social Sciences. <https://search.proquest.com/docview/2047378364?accountid=10267>

Jordan, J. L., & Elnagheeb, A. H. (1991). Public perceptions of food safety. *Journal of Food Distribution Research*, *22*(3), 13–22.

Kaiser, H. M., Scherer, C. W., & Barbano, D. M. (1992). Consumer perceptions and attitudes towards bovine somatotropin. *Northeastern Journal of Agricultural and Resource Economics*, *21*(1), 10–20.

Kennedy, D. M. (2007). *An economic analysis of the human health impacts of antibiotic use in food animal production and the demand for antibiotic -free meat* (304901178) [Ph.D., University of California, Davis]. ABI/INFORM Collection. <https://search.proquest.com/docview/304901178?accountid=10267>

Knowlton, K., & von Keyserlingk, M. (2018). To treat or not to treat: Public attitudes on the therapeutic use of antibiotics in the dairy industry. *Journal of Animal Science*, *96*, 502–503.

Köhler, F., & Wickenhäuser, M. (2001). *Strategies to address consumer concerns about animal welfare—Report on focus groups in Germany*.

Larson, R. B. (2019). Examining prospective buyer attitudes toward four food product traits. *British Food Journal*, *121*(8), 1936–1950. ABI/INFORM Collection. <https://doi.org/10.1108/BFJ-10-2018-0654>

Lee, T. L., & Thomson, D. U. (2016). A survey to describe beef producer opinions on antibiotic use and consumer perceptions of antibiotics in the beef industry. *Bovine Practitioner*, *50*(1), 58–63.

Leila Hamzaoui Essoussi, & Zahaf, M. (2009). Exploring the decision-making process of Canadian organic food consumers: Motivations and trust issues. *Qualitative Market Research*, *12*(4), 443–459. ABI/INFORM Collection. <https://doi.org/10.1108/13522750910993347>

Li, J., & Hooker, N. H. (2009). Documenting food safety claims and their influence on product prices. *Agricultural and Resource Economics Review*, *38*(3), 311–323.

López-Mas, L., Claret, A., Reinders, M. J., Banovic, M., Krystallis, A., & Guerrero, L. (2021). Farmed or wild fish? Segmenting European consumers based on their beliefs. *Aquaculture*, *532*, 735992. <https://dx.doi.org/10.1016/j.aquaculture.2020.735992>

Lusk, J. L. (2019). Consumer perceptions of ‘natural’ foods. *Food Technology* 73(7), 42-46.

Lusk, J. L., Foster, K., & Nilsson, T. (2007). Public Preferences and Private Choices: Effect of Altruism and Free Riding on Demand for Environmentally Certified Pork [electronic resource]. *Environmental & Resource Economics*, *36*(4), 499–521. agr.

Lusk, J. L., Norwood, F. B., & Pruitt, J. R. (2006). Consumer demand for a ban on antibiotic drug use in pork production. *American Journal of Agricultural Economics*, *88*(4), 1015–1033. <https://doi.org/10.1111/j.1467-8276.2006.00913.x>

Mannion, M., & Cowan, C. (1998). Beef safety concerns of consumers in Ireland. *Farm & Food*, *8*(4), 10–12.

McCarthy, M. B., & Henson, S. J. (2004). Irish consumer perceptions of meat hazards and use of extrinsic information cues. *Acta Agricultura Scandinavica. Section C, Economy*, *1*(2), 99–107. <https://doi.org/10.1080/16507540410035036>

McKendree, Melissa G. S., Widmar, N. O., Ortega, D. L., & Foster, K. A. (2013). Consumer Preferences for Verified Pork-Rearing Practices in the Production of Ham Products. *Journal of Agricultural and Resource Economics*, *38*(3), 397–417.

McKendree, Melissa Gale Short. (2013). *Exploring consumer preferences for animal care and rearing practices across species and products* (1490789063) [M.S., Purdue University]. ProQuest Dissertations & Theses Global: Social Sciences. <https://search.proquest.com/docview/1490789063?accountid=10267>

Mørkbak, M. R., Christensen, T., & Gyrd-Hansen, D. (2010). Consumer preferences for safety characteristics in pork. *British Food Journal*, *112*(7), 775–791. ABI/INFORM Collection. <https://doi.org/10.1108/00070701011058299>

Morkbak, M. R., Christensen, T., Gyrd-Hansen, D. E., & Olsen, S. B. (2009). Is Embedding Entailed in Consumer Valuation of Food Safety Characteristics? *IDEAS Working Paper Series from RePEc*. ABI/INFORM Collection. <https://search.proquest.com/docview/1697471269?accountid=10267>

Muringai, V., & Goddard, E. (2011). Canadian consumers’ consumption of pork and eggs: Do the same factors influence frequency of purchase? *Banff Pork Seminar Proceedings 2011*, A33.

Muringai, Violet. (2017). Trust, Perceptions, Intentions and Behaviour in Meat Consumption. *Journal of Food Distribution Research*, *48*(1), 8–9. bth.

Muringai, Violet, Goddard, E., Bruce, H., Plastow, G., & Ma, L. (2017). Trust and Consumer Preferences for Pig Production Attributes in Canada. *Canadian Journal of Agricultural Economics*, *65*(3), 477–514. ABI/INFORM Collection. <https://doi.org/10.1111/cjag.12138>

Muringai, Violet, & Goddard, E. W. (2010). Canadian Consumer Concerns About Food Safety Issues and Confidence in Food Products: Comparison of Beef and Pork. *IDEAS Working Paper Series from RePEc*. ABI/INFORM Collection. <https://search.proquest.com/docview/1697473160?accountid=10267>

Nayga, R. M. Jr. (1996). Sociodemorgraphic influences on consumer concern for food safety: The case of irradiation, antibiotic, hormones, and pesticides. *Review of Agricultural Economics*, *18*(3), 467–475. agr.

New National Survey From American Humane Association Shows Consumers Very Concerned About Animal Welfare And Want Humanely Raised Foods: Foster Farms Continues Commitment to Animal Welfare. (2013, September 18). *PR Newswire*. ABI/INFORM Collection. <https://search.proquest.com/docview/1433356506?accountid=10267>

Next to Knowing Your Farmer, the Organic Label is the Best Way to Trust Your Food: Single-Trait Label Claims Create Consumer Confusion and Misunderstanding of Organics. (2017, December 11). *NASDAQ OMX’s News Release Distribution Channel*. ABI/INFORM Collection. <https://search.proquest.com/docview/1974973365?accountid=10267>

Nilsson, T. K. H. (2005). *Product and process certification in imperfectly competitive markets: An application to the United States pork markets* (305388052) [Ph.D., Purdue University]. ABI/INFORM Collection. <https://search.proquest.com/docview/305388052?accountid=10267>

Nuppenau, E. A. (2015). Mitigating production practices and antibiotics use in meat industries prone to economies of scale by institutional novelties, marketing and voluntary actions. *Journal of Food Research*, *4*(3), 162–181.

Olynk, N. J., Tonsor, G. T., & Wolf, C. A. (2010). Consumer willingness to pay for livestock credence attribute claim verification. *Journal of Agricultural and Resource Economics*, *35*(2), 261–280.

Olynk, Nicole J., & Ortega, D. L. (2013). Consumer preferences for verified dairy cattle management practices in processed dairy products. *Food Control*, *30*(1), 298–305. <https://doi.org/10.1016/j.foodcont.2012.07.030>

Olynk Widmar, N. J., Byrd, E. S., Wolf, C. A., & Acharya, L. (2016). Health Consciousness and Consumer Preferences for Holiday Turkey Attributes. *Journal of Food Distribution Research*, *47*(2), 83–97. bth.

O’Neill, F. K. (1993). Dietary concerns of Irish consumers. *Farm & Food*, *3*(1), 10–11.

Ortega, D. L., Olynk Widmar, N. J., & Wang, H. H. (2014). Aquaculture imports from Asia: An analysis of U.S. consumer demand for select food quality attributes [electronic resource]. *Agricultural Economics*, *45*(5), 625–634. agr.

Ortega, D. L., Wang, H. H., & Widmar, N. J. O. (2015). Effects of media headlines on consumer preferences for food safety, quality and environmental attributes. *Australian Journal of Agricultural and Resource Economics*, *59*(3), 433–445. <https://doi.org/10.1111/1467-8489.12097>

Parsons, E. L. (2019). Poultry Labeling: Knowledge, Perceptions, and Preferences among Adolescents. Master’s Thesis, University of Arkansas [https://scholarworks.uark.edu/etd/3399](https://scholarworks.uark.edu/etd/3399?utm_source=scholarworks.uark.edu/etd/3399&utm_medium=PDF&utm_campaign=PDFCoverPages)

Picardy, J. A. (2015). *Niche Pork: Exploring opportunities for alternative swine production and marketing* (1723023720) [Ph.D., Tufts University, Gerald J. and Dorothy R. Friedman School of Nutrition Science and Policy]. Agricultural & Environmental Science Dissertations; ProQuest Dissertations & Theses Global: Science & Technology; ProQuest Dissertations & Theses Global: Social Sciences. <https://search.proquest.com/docview/1723023720?accountid=10267>

Picardy, J. A., Cash, S. B., & Peters, C. (2020). Uncommon Alternative: Consumers’ Willingness to Pay for Niche Pork Tenderloin in New England. *Journal of Food Distribution Research*, *51*(856-2020-1663), 61-91.

Pulcini, D., Franceschini, S., Buttazzoni, L., Giannetti, C., & Capoccioni, F. (2020). Consumer preferences for farmed seafood: An Italian case study. *Journal of Aquatic Food Product Technology*, *29*(5), 445-460. <https://dx.doi.org/10.1080/10498850.2020.1749201>

Riordan, N., Cowan, C., & McCarthy, M. (2002). Safety of Irish beef—Concerns, awareness and knowledge of Irish consumers. *Journal of Food Safety*, *22*(1), 1–15. <https://doi.org/10.1111/j.1745-4565.2002.tb00326.x>

Ritter, G. D., Acuff, G. R., Bergeron, G., Bourassa, M. W., Chapman, B. J., Dickson, J. S., Opengart, K., Salois, M. J., Singer, R. S., & Storrs, C. (2019). Antimicrobial-resistant bacterial infections from foods of animal origin: Understanding and effectively communicating to consumers. *Annals of the New York Academy of Sciences*, *1441*(1), 40–49. <https://doi.org/10.1111/nyas.14091>

Roembke, J. (2018). Marketing-driven poultry production’s real feed costs. *WATT PoultryUSA*, *19*(2), 22-N.PAG. bth.

Roheim, C. A., Durham, C. A., & Sudhakaran, P. O. (2012). Certification of shrimp and salmon for best aquaculture practices: Assessing consumer preferences in rhode island [electronic resource]. *Aquaculture Economics & Management*, *16*(3), 266–286. agr.

Saitone, T. L., Sexton, R. J., & Sumner, D. A. (2015). What Happens When Food Marketers Require Restrictive Farming Practices? *American Journal of Agricultural Economics*, *97*(4), 1021–1043. <https://doi.org/10.1093/ajae/aav021>

Sato, P., Hotzel, M. J., & von Keyserlingk, M. A. G. (2017). American Citizens’ Views of an Ideal Pig Farm. *Animals*, *7*(8), 64. <https://doi.org/10.3390/ani7080064>

Sloan, A. E. (1995). Feeling safe about food safety. *Food Technology*, *49*(6).

Smallwood, D. M. (1989). Consumer demand for safer foods. *National Food Review*, *12*(3), 9–11.

Smith, J. M., & Ferris, T. A. (2016). Breakfast on the farm, an educational farm tour, improves consumer trust in animal care, food safety, and modern conventional dairy production. *Journal of Animal Science*, *94*(Suppl. 5), 274–275. <https://doi.org/10.2527/jam2016-0580>

Smith, R. A., Zhu, X., Shartle, K., Glick, L., & M’ikanatha, N. M. (2017). Understanding the Public’s Intentions to Purchase and to Persuade Others to Purchase Antibiotic-Free Meat. *Health Communication*, *32*(8), 945–953. <https://doi.org/10.1080/10410236.2016.1196415>

Sonntag, W. I., Spiller, A., & von Meyer-Hofer, M. (2019). Discussing modern poultry farming systems-insights into citizen’s lay theories. *Poultry Science*, *98*(1), 209–216. <https://doi.org/10.3382/ps/pey292>

Steiner, B. E., & Yang, J. (2010). How do U.S. and Canadian consumers value credence attributes associated with beef labels after the North American BSE crisis of 2003? *International Journal of Consumer Studies*, *34*(4), 449–463. ABI/INFORM Collection. <https://doi.org/10.1111/j.1470-6431.2010.00889.x>

Stolz, H., Hamm, U., Janssen, M., & Stolze, M. (2011). Preferences and determinants for organic, conventional and conventional-plus products – The case of occasional organic consumers [electronic resource]. *Food Quality and Preference*, *22*(8), 772–779. agr.

Tait, P. R., Rutherford, P., Driver, T., XueDong, L., Saunders, C. M., & Dalziel, P. C. (2018). Consumer insights and willingness to pay for attributes: New Zealand beef products in California, USA. *Research Report - Agribusiness & Economics Research Unit, Lincoln  University*, *348*, xii + 78 pp.

Thilmany, D. D., Umberger, W. J., & Ziehl, A. R. (2006). Strategic market planning for value-added natural beef products: A cluster analysis of Colorado consumers. *Renewable Agriculture and Food Systems*, *21*(3), 192–203. <https://doi.org/10.1079/RAF2005143>

Tong, B. (2011). *Canadian organic and raised without antibiotics pork consumer characteristics* (859606670) [M.Sc., University of Guelph (Canada)]. ABI/INFORM Collection. <https://search.proquest.com/docview/859606670?accountid=10267>

Ubilava, D., Foster, K. A., Lusk, J. L., & Nilsson, T. (2010). Effects of income and social awareness on consumer WTP for social product attributes. *Technological Forecasting and Social Change*, *77*(4), 587. ABI/INFORM Collection. <https://doi.org/10.1016/j.techfore.2009.02.002>

Umberger, W. J., Boxall, P. C., & Lacy, R. C. (2009). Role of credence and health information in determining US consumers’ willingness-to-pay for grass-finished beef. *Australian Journal of Agricultural & Resource Economics*, *53*(4), 603–623. bth.

Upah, K. M. (2016). *Generational perceptions of beef credence labels in the United States* (1830772016) [M.S., Southern Illinois University at Carbondale]. Agricultural & Environmental Science Dissertations; ProQuest Dissertations & Theses Global: Science & Technology. <https://search.proquest.com/docview/1830772016?accountid=10267>

Van Loo, E., Caputo, V., Nayga, R. M. J., Meullenet, J.-F., Crandall, P. G., & Ricke, S. C. (2010). Effect of organic poultry purchase frequency on consumer attitudes toward organic poultry meat. *Journal of Food Science*, *75*(7), S384-397. <https://doi.org/10.1111/j.1750-3841.2010.01775.x>

Veeman, M., & Yu, L. (2007). Investigating changes in Canadian consumers’ food safety concerns, 2003 and 2005. *Rural Economy Project Report - Department of Rural Economy, University  of Alberta*, *07–02*, 29 pp.

Viegas, I., Santos, J. M. L., & Fontes, M. A. (2011). Joint Production of Safer, Cleaner and Animal Friendlier Beef: Do Consumers Join it Too? - Insights from Focus Groups. *IDEAS Working Paper Series from RePEc*. ABI/INFORM Collection. <https://search.proquest.com/docview/1697475693?accountid=10267>

Warren, V. A., Jennings, G. E., & Hillers, V. N. (1990). Beliefs about food supply safety: A study of cooperative extension clientele. *Journal of the American Dietetic Association*, *90*(5), 713–714. agr.

Webb, M. J., Pendell, D. L., Harty, A. A., Salverson, R. R., Rotz, C. A., Underwood, K. R., Olson, K. C., & Blair, A. D. (2017). Beef production technology and its effect on consumer preference. *National Provisioner*, *231*(12), 18–19. ABI/INFORM Collection.

Wemette, M., Safi, A. G., Wolverton, A. K., Beauvais, W., Shapiro, M., Moroni, P., ... & Ivanek, R. (2021). Public perceptions of antibiotic use on dairy farms in the United States. *Journal of Dairy Science*, *104*(3), 2807-2821. <https://dx.doi.org/10.3168/jds.2019-17673>

Widmar, N. O., Morgan, C. J., Wolf, C. A., Yeager, E. A., Dominick, S. R., & Croney, C. C. (2017). US resident perceptions of dairy cattle management practices. *Agricultural Sciences*, *8*(7), 645–656. <https://doi.org/10.4236/as.2017.87049>

Wolf, C. A., Tonsor, G. T., McKendree, M. G. S., Thomson, D. U., & Swanson, J. C. (2016). Public and farmer perceptions of dairy cattle welfare in the United States. *Journal of Dairy Science*, *99*(7), 5892–5903. <https://doi.org/10.3168/jds.2015-10619>

Wolf, I. D. (1992). Critical issues in food safety, 1991-2000. *Food Technology*, *46*(1), 64, 66, 68–70.

Zhang, Y. (2019). *Consumer willingness-to-pay for antibiotic claims in ground beef with the implementation of a ban on the use of antibiotics for growth promotion in feed* (Doctoral dissertation, University of Delaware).

Zingg, A., & Siegrist, M. (2012). People’s willingness to eat meat from animals vaccinated against epidemics. *Food Policy*, *37*(3), 226–231. <https://doi.org/10.1016/j.foodpol.2012.02.001>
