# Supplementary material for: Consumer perceptions of antimicrobial use in animal husbandry: A scoping review
Source: PLoS One. 2021 Dec 8;16(12):e0261010. doi: 10.1371/journal.pone.0261010 (PMC8654221; doi:10.1371/journal.pone.0261010)
Supplement: Attachment — Submitted filename: PONE-D-21-21650_reviewer.pdf [file pone.0261010.s003.pdf]

PLOS ONE

Consumer perceptions of antimicrobial use in animal agriculture in the United States, Canada, and the European Union: A scoping review

--Manuscript Draft--

**Manuscript Number:**	PONE-D-21-21650
**Article Type:**	Research Article
**Full Title:**	Consumer perceptions of antimicrobial use in animal agriculture in the United States, Canada, and the European Union: A scoping review
**Short Title:**	A Scoping review of consumer perceptions of antimicrobial use in animal agriculture
**Corresponding Author:**	David Lansing<br>University of Maryland Baltimore County<br>Baltimore, MD UNITED STATES
**Keywords:**	Economics; Agricultural Economics; Environmental Economics; Bioeconomics; health economics; Information economics; Microeconomics; Household economics; Economic analysis; Cost-benefit analysis; Cost-effectiveness analysis; Risk-benefit analysis; Foodborne diseases
**Abstract:**	Antimicrobial use in animal agriculture is often perceived to play a role in the emerging threat of antimicrobial resistance. Increased consumer awareness of this issue places pressure on animal agriculture to adopt policies to reduce or eliminate antimicrobial use. We use a scoping review methodology to assess research on consumer perceptions of antimicrobial drugs in meat products in the United States, Canada, or the European Union. Evaluating peer reviewed and grey literature, we included studies for assessment if they met these topical and geographic requirements, involved primary data collection, and were originally published in English. Our screening process identified 125 relevant studies. Three reviewers jointly developed a data charting form and independently charted the contents of the studies. Of the 106 studies that directly measured consumer concern, 77.4% found that consumers were concerned about antimicrobial use in meat production. A minority of studies (29.6% of all studies) queried why consumers hold these views. These studies found human health and animal welfare were the main reasons for concern. Antimicrobial resistance rarely registered as an explicit reason for concern. A smaller group of studies (23.2%) measured the personal characteristics of consumers that expressed concerned about antimicrobials. The most common and consistent features of these consumers were gender, age, income, and education. Overall, studies tended to be dominated by either willingness-to-pay studies or likert scale questionnaires (73.6% of all studies). The popularity of these methods may have contributed to the relative lack of studies that characterized worried consumer demographics or reasons for their perspectives. We recommend more qualitative research into consumer views on this topic, which may better elucidate consumer decision-making and mentality. In addition, more research into the difference between what consumers claim is of concern and their ultimate purchasing decisions would be especially valuable.
**Order of Authors:**	Jaime R Barrett
	Gabriel K Innes
	Kelly A Johnson
	Guillaume Lhermie
	Renata Ivanek
	Amelia Greiner Safi
	David Lansing, Ph.D
**Additional Information:**	
**Question**	**Response**
**Financial Disclosure**	JRB, GKI, GL, RI, AGS, DL received funds from the United States Department of

Powered by Editorial Manager® and ProduXion Manager® from Aries Systems Corporation

Enter a financial disclosure statement that describes the sources of funding for the work included in this submission. Review the submission guidelines for detailed requirements. View published research articles from *PLOS ONE* for specific examples.<br><br>This statement is required for submission and **will appear in the published article** if the submission is accepted. Please make sure it is accurate.<br><br>**Unfunded studies**<br>Enter: *The author(s) received no specific funding for this work.*<br><br>**Funded studies**<br>Enter a statement with the following details:<br>• Initials of the authors who received each award<br>• Grant numbers awarded to each author<br>• The full name of each funder<br>• URL of each funder website<br>• Did the sponsors or funders play any role in the study design, data collection and analysis, decision to publish, or preparation of the manuscript?<br>• **NO** - Include this sentence at the end of your statement: *The funders had no role in study design, data collection and analysis, decision to publish, or preparation of the manuscript.*<br>• **YES** - Specify the role(s) played.<br><br>* typeset	Agriculture, National Institute for Food and Agriculture grant # 2019-67017-29114.<br><br>JRB, DL received funds from USDA-NIFA grant #2018-68003-27467<br>https://nifa.usda.gov/<br>The funders had no role in the study design, data collection and analysis, decision to publish, or preparation of the manuscript.
**Competing Interests**<br><br>Use the instructions below to enter a competing interest statement for this submission. On behalf of all authors, disclose any competing interests that could be perceived to bias this work—acknowledging all financial support and any other relevant financial or non-financial competing interests.<br><br>This statement is required for submission and **will appear in the published article** if	The authors have declared that no competing interests exist.

the submission is accepted. Please make sure it is accurate and that any funding sources listed in your Funding Information later in the submission form are also declared in your Financial Disclosure statement.<br><br>View published research articles from *PLOS ONE* for specific examples.<br><br>**NO authors have competing interests**<br><br>Enter: *The authors have declared that no competing interests exist.*<br><br>**Authors with competing interests**<br><br>Enter competing interest details beginning with this statement:<br><br>*I have read the journal's policy and the authors of this manuscript have the following competing interests: [insert competing interests here]*<br><br>* typeset	
**Ethics Statement**<br><br>Enter an ethics statement for this submission. This statement is required if the study involved:<br><br>• Human participants<br>• Human specimens or tissue<br>• Vertebrate animals or cephalopods<br>• Vertebrate embryos or tissues<br>• Field research<br><br>Write "N/A" if the submission does not require an ethics statement.<br><br>General guidance is provided below. Consult the submission guidelines for detailed instructions. **Make sure that all information entered here is included in the Methods section of the manuscript.**	N/A

**Format for specific study types**<br><br>**Human Subject Research (involving human participants and/or tissue)**<br>• Give the name of the institutional review board or ethics committee that approved the study<br>• Include the approval number and/or a statement indicating approval of this research<br>• Indicate the form of consent obtained (written/oral) or the reason that consent was not obtained (e.g. the data were analyzed anonymously)<br><br>**Animal Research (involving vertebrate animals, embryos or tissues)**<br>• Provide the name of the Institutional Animal Care and Use Committee (IACUC) or other relevant ethics board that reviewed the study protocol, and indicate whether they approved this research or granted a formal waiver of ethical approval<br>• Include an approval number if one was obtained<br>• If the study involved *non-human primates*, add *additional details* about animal welfare and steps taken to ameliorate suffering<br>• If anesthesia, euthanasia, or any kind of animal sacrifice is part of the study, include briefly which substances and/or methods were applied<br><br>**Field Research**<br><br>Include the following details if this study involves the collection of plant, animal, or other materials from a natural setting:<br>• Field permit number<br>• Name of the institution or relevant body that granted permission	
**Data Availability**<br><br>Authors are required to make all data underlying the findings described fully available, without restriction, and from the time of publication. PLOS allows rare exceptions to address legal and ethical concerns. See the PLOS Data Policy and FAQ for detailed information.	Yes - all data are fully available without restriction

A Data Availability Statement describing where the data can be found is required at submission. Your answers to this question constitute the Data Availability Statement and **will be published in the article**, if accepted.<br><br>**Important:** Stating 'data available on request from the author' is not sufficient. If your data are only available upon request, select 'No' for the first question and explain your exceptional situation in the text box.<br><br>Do the authors confirm that all data underlying the findings described in their manuscript are fully available without restriction?	
**Describe where the data may be found in full sentences. If you are copying our sample text, replace any instances of XXX with the appropriate details.**<br><br>• If the data are **held or will be held in a public repository**, include URLs, accession numbers or DOIs. If this information will only be available after acceptance, indicate this by ticking the box below. For example: *All XXX files are available from the XXX database (accession number(s) XXX, XXX.*).<br>• If the data are all contained **within the manuscript and/or Supporting Information files**, enter the following: *All relevant data are within the manuscript and its Supporting Information files.*<br>• If neither of these applies but you are able to provide **details of access elsewhere**, with or without limitations, please do so. For example:<br><br>*Data cannot be shared publicly because of [XXX]. Data are available from the XXX Institutional Data Access / Ethics Committee (contact via XXX) for researchers who meet the criteria for access to confidential data.*<br><br>*The data underlying the results presented in the study are available from (include the name of the third party*	The charting data that is the basis of our analysis can be found at: https://osf.io/27pyw/

and contact information or URL).<br>• This text is appropriate if the data are owned by a third party and authors do not have permission to share the data.<br><br>* typeset	
Additional data availability information:	

Powered by Editorial Manager® and ProduXion Manager® from Aries Systems Corporation

Manuscript

Click here to access/download;Manuscript;AMR Scoping_Final_7121.docx

Consumer perceptions of antimicrobial use in animal agriculture in the United States, Canada, and the European Union: A scoping review

Authors

Jaime R. Barrett
Contributed equally to this work with Gabriel K. Innes
Roles: Conceptualization, Data Curation, Formal Analysis, Investigation, Writing-original, Writing-review and editing
Affiliation: Department of Geography and Environmental Systems, University of Maryland Baltimore County, Baltimore, MD, USA[†]

Gabriel K. Innes
Contributed equally to this work with Jaime R. Barrett
Roles: Conceptualization, Data Curation, Formal Analysis, Investigation, Writing-original, Writing-review and editing
Affiliation: Department of Biostatistics and Epidemiology, Rutgers School of Public Health, Piscataway, NJ, USA

Kelly A. Johnson
Roles: Data curation, Investigation, Methodology, Project Administration, Writing-original, Writing-review and editing
Affiliation: Flower-Sprecher Veterinary Library, College of Veterinary Medicine, Cornell University, Ithaca, NY, USA

Guillaume Lhermie
Roles: Conceptualization, Formal Analysis, Investigation, Writing – original, Writing – review and editing
Affiliation: Department of Production Animal Health, University of Calgary, Calgary, T2N 4Z6, Canada; CIRAD, UMR ASTRE, Montpellier, France, ASTRE, CIRAD, INRAE, Univ Montpellier, Montpellier, Université de Toulouse, ENVT, 31300 Toulouse, France

Renata Ivanek
Roles: Conceptualization, Investigation, Writing – review and editing
Affiliation: Department of Population Medicine and Diagnostic Sciences, College of Veterinary Medicine, Cornell University, Ithaca, NY, USA

Amelia Greiner Safi
Roles: Conceptualization, Investigation, Writing – review & editing

Affiliation: Department of Public and Planetary Health, College of Veterinary Medicine & Department of Communication, College of Agriculture and Life Sciences Cornell University, Ithaca, NY, USA

David Lansing
Roles: Conceptualization, Data Curation, Investigation, Formal analysis, Project Administration, Writing – original draft, Writing – review & editing
Affiliation: Department of Geography and Environmental Systems, University of Maryland Baltimore County, Baltimore, MD, USA

ABSTRACT:

Antimicrobial use in animal agriculture is often perceived to play a role in the emerging threat of antimicrobial resistance. Increased consumer awareness of this issue places pressure on animal agriculture to adopt policies to reduce or eliminate antimicrobial use. We use a scoping review methodology to assess research on consumer perceptions of antimicrobial drugs in meat products in the United States, Canada, or the European Union. Evaluating peer reviewed and grey literature, we included studies for assessment if they met these topical and geographic requirements, involved primary data collection, and were originally published in English. Our screening process identified 125 relevant studies. Three reviewers jointly developed a data charting form and independently charted the contents of the studies. Of the 106 studies that directly measured consumer concern, 77.4% found that consumers were concerned about antimicrobial use in meat production. A minority of studies (29.6% of all studies) queried why consumers hold these views. These studies found human health and animal welfare were the main reasons for concern. Antimicrobial resistance rarely registered as an explicit reason for concern. A smaller group of studies (23.2%) measured the personal characteristics of consumers that expressed concerned about antimicrobials. The most common and consistent features of these consumers were gender, age, income, and education. Overall, studies tended to be dominated by either willingness-to-pay studies or likert scale questionnaires (73.6% of all studies). The popularity of these methods may have contributed to the relative lack of studies that characterized worried consumer demographics or reasons for their perspectives. We recommend more qualitative research into consumer views on this topic, which may better elucidate consumer decision-making and mentality. In addition, more research into the difference between

what consumers claim is of concern and their ultimate purchasing decisions would be especially valuable.

INTRODUCTION

The rise of antimicrobial resistant organisms threaten human and animal health (Robinson et al. 2016). In livestock production systems, antimicrobials have been used for prevention and treatment of disease and, in many countries, growth promotion (Oliver et al., 2011; Landers, 2012). Antimicrobial use in animal agriculture has been linked to antimicrobial resistant bacterial infections in humans (Innes, et al. 2020). To address public concern about antimicrobial resistance, regulation has been promulgated to limit the use of certain drugs in animal agriculture (Kirchhelle, 2018). A recent amendment in 2017 to the Veterinary Feed Directive of the United States Department of Agriculture's (USDA) Animal Drug Availability Act of 1996 changed drug use allowances in U.S. animal agriculture industries. This amendment prohibits the use of medically important antimicrobials in food-producing animals for growth promotion or to improve feed efficiency, and requires approval from the overseeing veterinarian for antimicrobials that are administered via feed and water (FDA 2015, 2017, 2019). In addition to this federal regulation, state governments such as California (California SB27, 2015) and Maryland (Pinscky et al, 2017) have implemented laws in 2018 that also restrict antimicrobial use in agriculture. As with the VFD, the effectiveness of these bills has yet to be assessed.

Governmental regulatory efforts may prove to be an important step in decreasing antimicrobial resistance development in the animal agriculture, however, private industry standards are

increasingly the impetus for progress in the agri-food system (Busch and Bain 2004). Many agricultural standards are voluntary and put forth by private companies and trade associations (e.g., national dairy associations) to avoid further government regulation (Busch and Bain 2004, Jones and Pawlinger 2017). These shifts are also driven by the need to maintain their consumer base in a saturated market (Nestle 2002) and therefore attempt to address consumer demand for safe food of a uniform quality that is produced under conditions consumers can support (Busch and Bain 2004, Jones and Pawlinger 2017). For example, large animal product purchasers, such as McDonalds and public-school systems, have committed to using "antibiotic free" animal products (Polansek 2014; McDonald's Global Vision for Antibiotic Stewardship in Food Animals, 2017). Consumers cite human health, animal welfare, and environmental sustainability as reasons for their concern about antimicrobial use in animal agriculture (Foundation IFIC, 2018). Consumer attitudes may also reflect confusion about modern production practices. For example, some consumers purchase "raised without antibiotics" animal products because of their concerns for animal welfare (Goddard et al., 2017; Karavoilias et al., 2017). However, these consumers may not understand that antimicrobials are necessary for the prevention and treatment of diseases in animals, and thus a complete ban could lead to increased animal suffering in cases of clinical infections if they are withheld (Oliver et al., 2011; Karavolias et al., 2017).

Despite potential consumer confusions about the role of antimicrobials in animal agriculture, such perceptions are important drivers of animal husbandry practices across the wider commodity chain. The adoption of practices market products with labels such as "no antibiotics ever" in the poultry industry, for example, exerts downward pressure on the production practices of broiler integrators (Bowman et al. 2016). Similar consumer driven pressures have been noted

across other animal production industries as well (eg. Singer et al. 2019). In short, the increasing prevalence of "antibiotic free" labels on food, and emerging evidence that consumers will pay more for meat with this label, mean that the consumers influence the governance of wider food systems.

While research on consumer preferences for meat purchase and consumption is explored in the scientific literature, the salience of antimicrobial use in food production calls for a closer examination of the scientific evidence on this topic. To the best of our knowledge, no review has investigated consumer perceptions of antimicrobial use in animal agriculture; we fill this gap with a scoping review. We aim to summarize the extant research on this topic, identify research areas that are both well-studied and ignored, and understand what consumers see as the risks and benefits of antimicrobial use in animal agriculture. Further, we identify the methods used to assess consumer perception in order to gauge existing methodological gaps in the literature.

MATERIALS AND METHODS

This review was completed in compliance with the guidelines outlined in the PRISMA Extension for Scoping Reviews (Tricco et al. 2018). The review team was composed of experts in the field (Redacted for Review), a research librarian (Redacted for Review), and students (Redacted for Review).

RESEARCH QUESTION AND DEFINITIONS

This review aims to identify and describe peer-reviewed and grey literature relevant to the research question: "What are consumer perceptions concerning antimicrobial use in animal

agriculture in the United States, Canada, and the European Union?" and utilizes the following definitions.

Reference needed for the text from Line 147 to Line 165

Consumer perceptions and attitudes: Consumers are defined as individuals who purchase food. Of particular interest to this review are consumers who purchase animal-based products for personal or familial consumption or consumers who choose not to purchase animal-based products and their reasoning. Perception encompasses awareness, understanding and interpretation of an individual's surroundings. Attitude includes, but is not limited to, one’s thoughts, feelings, beliefs, and willingness to pay for food. In combination this review will assess the level of awareness and understanding of general audiences in regard to antimicrobials in animal products and animal agriculture.

Antimicrobials: Antimicrobials are defined here as drugs that are administered to patients to treat and/or prevent infection, illness, and/or other health problems resulting from exposure to microbial organisms. These can include antibiotics, antifungals, antiprotozoals, and antivirals. For the purposes of this review we are interested in antimicrobials administered to maintain the health and well-being of agricultural animals raised for human consumption, of which antibiotics (i.e., drugs that target bacteria) are primarily used.

Animal agriculture: Animal agriculture is the husbandry of animals for consumption of their meat or other products. Animals included in this category are as follows: ruminants (cattle, sheep, goats, bison), pigs, poultry (chickens, turkeys, ducks), and fish (shellfish and finfish).

A protocol for this review was registered on the Open Science Framework (osf.io) on August 8, 2019, and can be located at https://osf.io/rp9ak/. An amendment was made at the initiation of full text screening and was uploaded on December 23, 2020, and can be located at https://osf.io/mcd93/.

SEARCH STRATEGY, DATABASES, AND GREY LITERATURE SOURCES

A comprehensive search was developed for CAB Abstracts and Global Health (CABI) using search terms related to consumer perceptions, antimicrobials, and animal agriculture. The search was translated and run in ABI/Inform (ProQuest), AGRICOLA (EBSCOhost), BIOSIS Citation Index (Clarivate Analytics), Business Source Complete (EBSCOhost), FSTA/Food Science and Technology Abstracts (Clarivate Analytics), Medline (PubMed), ProQuest Dissertations and Theses Global (ProQuest), VetMed Resource (CABI), and Web of Science Core Collection (Clarivate Analytics). Searches took places in two rounds: and initial search, and an updated search. The first round of searches were executed on August 14, 2019, without date or language restrictions. Search strategy and number of results for each database are found in Appendix A. Grey literature sources were searched between August 24, 2019 and September 24, 2019. Publications and factsheets were manually searched in: Agriculture and Agri-Food Canada; Canadian Antimicrobial Resistance Surveillance System; Centers for Disease Control and Prevention (CDC) Antibiotic/Antimicrobial Resistance Reports and Publications; Environmental Working Group; European Commission; European Food Safety Authority; Food and Agriculture Organization (FAO) of the United Nations; Food and Drug and Administration of the United States (FDA) Antimicrobial Resistance Information from FDA; FDA Guidance Documents; Pew Charitable Trusts Antibiotic Resistance Project; U.S. Department of Agriculture (USDA) Economic Research Service; and World Health Organization (WHO). Links, search strategies,

and number of results for each grey literature source are found in our registered protocol. A second round of searches was undertaken in May 2021. On May 10, 2021 a second round of searches were undertaken, without language restrictions, but data restricted from August 14, 2019 to May 10, 2021. From May 19-28, 2021, a second round of grey literature searches was also undertaken, utilitzing the same grey literature databases as stated above. Documentation of search terms and databases used for the academic search is documented in Appendix A is available on the project's OSF page: https://osf.io/p82fg/
Documentation of search terms and databases used for the grey literature searches is documented in Appendix B, and is available on the project's OSF page: https://osf.io/frxsw/

CITATION MANAGEMENT

References returned from all database and grey literature searches were imported or manually entered into Zotero citation management software (Version 5.0.73). Following deduplication in Zotero, the remaining records were imported to the screening software Covidence (covidence.org), where additional duplicates were identified. The remaining records were eligible for inclusion in the review.

STUDY SELECTION AND SCREENING

Studies were considered eligible for inclusion in this review if they: (1) include reference to antimicrobial use in food animals, (2) include consumer viewpoints about antimicrobial use in food animals, (3) describe studies in the United States, Canada, or the European Union, (4) are originally published in English, and (5) describe primary data collection. Studies were excluded if they did not satisfy all inclusion criteria.

Each record was evaluated against the predetermined inclusion criteria by two independent reviewers at the level of title and abstract. Those records that were not eliminated at this stage were then considered by two independent reviewers at the full-text level. For both the title and abstract stage and full-text stage, conflicts were resolved either by consensus or by a third, independent reviewer.

Number of sources included at each stage of retrieval, screening, and data extraction, as well as reasons for exclusion at the full-text screening phase, are indicated in the PRISMA diagram (Figure 1). As prescribed for scoping reviews (Tricco et al. 2018, Arksey and O'Malley 2005), risk of source bias was not evaluated during consideration for inclusion.

DATA CHARTING AND ANALYSIS

Based on trends and concepts identified during screening, a list of relevant data categories was developed to guide data extraction. Each of the three main reviewers [Redacted for Review] extracted data from five papers to evaluate the list's comprehensiveness. Additional categories were added after this pre-testing, as well as during the extraction process when new trends were identified. One of the three main reviewers extracted data from each of the studies. Multiple discussions throughout this process were used to ensure consistency. The data from this charting process is available at: https://osf.io/27pyw/. This data includes charting from both the initial and the updated searches.

Extracted data includes: study type (qualitative or quantitative), publication source, author affiliation, publication date, country of study popuulation, number of participants, response rate, population selection criteria, product of study, data collection method, qualitative and quantitative models and associated analysis units (willingness to pay and Likert scale), specific

results about perceptions of antimicrobial use and several binary variables for statistical analysis. The extracted data were coded in anticipation of statistical analysis.

When developing the protocol for this review, we limited our scope to studies about the U.S., Canada, and the European Union (including the United Kingdom). We made this decision because these countries have similar regulatory environments and close trade associations. We also excluded texts written in non-English languages due a lack of reading proficiency among authors. Therefore, some otherwise relevant Canadian and European studies were excluded. Between the title and abstract stage and full text screening stage of this review, we further decided to exclude any texts that did not contain primary data collection (reflected in the amended protocol). As a result, most of the originally included news articles and opinion pieces became excluded. This decision was made in an effort to avoid bias as we could not ensure that all non-academic texts about this topic were captured. Several news articles with extractable data were included in the final analysis because they cited studies that were not otherwise captured through database and grey literature searches. Although our search strategy was comprehensive in its use of "antimicrobial" and the other associated terms listed above, extracted studies about consumer concern all focused on antibiotic use as opposed to antimicrobial use; and the term “antibiotic” was overwhelmingly used in these studies. For this reason, we use the more specific term "antibiotics" for the results and discussion sections.

To answer our proposed question we performed additional analysis on the studies that measured consumer concern. For manuscripts which utilized Likert scale surveys, studies were classified as finding that consumers were “concerned” if there was, on average, a higher than neutral level of agreement with a statement that expressed concern about antibiotic use. Conversely, Likert surveys that discovered a lower than neutral level of agreement for similar statements were

coded as finding that consumers were “not concerned.” Willingness-to-pay studies that showed consumers were willing to pay more for food with antibiotic-free traits (at a statistically significant level) were labeled as studies that showed consumers are “concerned.” Similarly, willingness-to-pay studies that failed to find consumers would pay more for antibiotic-free food were coded as having found consumers to be “not concerned.” Some studies found that consumers agreed with some concern-type statements while disagreeing with others; such studies were labeled as “mixed concern.”

Reasons for consumer concern were identified and each reason was given a unique identifier for analysis. For studies that investigated the characteristics of people who are concerned about antimicrobial use, demographics (e.g., gender, religion) determined to be statistically significant were tallied. Most studies that evaluated consumer characteristics concluded that multiple characteristics were associated with antibiotic use concerns. This resulted in more consumer characteristics identified than papers identifying such traits.

The coded spreadsheet of extracted data was imported into Stata (Version MP 16) to perform descriptive statistical analysis. Statistical tables including frequencies and percentages were generated to identify dominant categories for each extracted data type. More in-depth analysis of results was used in conjunction with frequency and percentage statistics to assess for gaps in the research.

RESULTS

Study selection and exclusion criteria are summarized by the PRISMA flow diagram illustrated in Figure 1. From the 3,560 citations imported for title and abstract screening, 368 were chosen for full text screening and 125 met inclusion criteria. Table 1 shows publication date ranges, study locations, and author affiliations for studies ultimately selected for inclusion. Due to the inclusion criterion of primary data collection, most of the relevant texts were published in academic journals (67.2%) with news articles a distant second (7.2%); the remaining 25.6% were a mix of other publication types, such as dissertations. Publications before 2009 comprise 30.4% of the sample, 27.9% were published between 2010 and 2015, and 42.4% were published between 2016 and 2021. The majority of research was conducted in the United States (54.4%). Canada (9.6%) and Germany (6.4%) were the next most commonly studied countries. Most studies (72%) were conducted solely by university researchers. Government researchers accounted for 5.6% of studies and industry researchers comprise another 6.4%.

Many animal agriculture products were investigated, with no single type dominating the body of literature (Table 2). The most frequently investigated single product categories are pork (15.2%) and beef (13.6%), poultry (10.4%), and dairy (10.4%). The most frequent product category is the generic category (24%), which includes studies that investigated “food,” “organic food,” “meats,” and/or other similarly broad categories. Multiple product studies were tied for the second most frequent category (15.2%) and included a range of product combinations from pork and eggs to dairy products and apples.

Studies often had multiple themes but those tallied in Table 2 were identified by reviewers as the primary focus of each study. We found 18 distinct research themes among which antibiotic

perception data could be assessed. Few publications (12.8%) had a central focus on consumer perceptions of antibiotics. More commonly, antibiotics were one of several consumer concerns that were measured in a study. Of the studies with a main focus on antibiotic use, dairy (n = 6) and beef (n = 4) were the most common, followed by pork (n = 2) (See Figure 2). Other core topics for studies include production characteristics (23.2%), food safety (16%), and credence claims/product attributes (10.4%). The production characteristics category includes any publication that focuses on agricultural practices and other aspects of production, e.g.,. rearing practices, conventional versus organic production, and other similar foci. The credence claims/product attributes category encompasses publications with a primary focus on perceptions of particular food characteristics, e.g., raised without antibiotics, natural, organic, and other labeled product attributes.

The publications under review were dominated by quantitative methods (82.4%; see Table 3). Qualitative methods—including interviews, focus groups, and document analysis—were used in 11.2% of the studies, and mixed quantitative/qualitative techniques were used in 6.4% of studies. Data collection was divided into five categories: surveys (56%), choice experiments (7.2%), qualitative methods (6.4%), document and literature analysis (6.4%), and mixed approaches (20.8%). Four studies (3.2%) did not identify their method of data collection. In terms of specific quantitative techniques, willingness-to-pay studies (34.4%) and Likert scale surveys (39.2%) were the most utilized techniques used to ascertain consumer perceptions.

Economics is the dominant field of research that investigated consumer attitudes and concerns with antibiotic use in animal agriculture, with 44.8% of the texts describing an economic or

marketing component of consumer perceptions. Of these papers, 17.9% did not collect original data and 12.5% had unclear or missing information. The remaining publications (69.6%) consisted of consumer surveys administered to a varying number of people (min: 154, max: 7795). These studies used a variety of econometric analyses; 14 studies used a choice experiment approach, three used different kinds of stated preference approach, and eight used econometric analyses without assessing consumer preferences. Other analysis methods were also used; 11studies reported only descriptive statistics and univariate or bivariate analysis, and the final four studies reported only qualitative information. Of these 56 economics-focused studies, 25% primarily focused on antibiotics. The other studies investigated antimicrobial use as a component of animal rearing or a characteristic of food products themselves. Additionally, the challenge of antimicrobial resistance, with regards to public health, was a particular source of concern with only one study (Dohle et al. 2013), which explored the environmental consequences of antimicrobial use and antimicrobial resistance development. Instead, antimicrobials were studied generally as a food safety issue, or with a set of other issues such as organic vs. conventional farming, animal welfare, and food quality. In most studies that utilized a willingness-to-pay model, people surveyed were willing to pay a premium for antibiotic-free products but this varied (between 0% and approximately 80%) depending on the geographic, social and cultural settings investigated.

CONSUMER CONCERN ABOUT ANTIBIOTICS

Research on consumer perceptions of antibiotic use in animal agriculture encompasses a wide variety of subjects, and researchers utilized several measurement techniques, which challenges

the ability to summarize findings among studies. Nevertheless, most studies found that consumer perceptions of antibiotic use exist along a spectrum. As described in the methods section, studies that gauged a level of concern about antibiotic use were coded as finding that consumers were “concerned about antibiotic use,” “not concerned about antibiotic use,” or had “mixed concern about antibiotic use.” A total of 84.8% of studies were able to be classified in this way. The remaining studies measured other aspects of consumers perceptions, such as whether they know what an antibiotic-free label means (eg. Abrams 2010; Nuppenau 2015).

Among the literature investigated, 65.6% of studies concluded that consumers were concerned with antibiotic use in food production, 8% were not concerned, and 11.2% showed mixed concern (see Table 4). Figure 2 summarizes the findings of studies that gauged consumer concern by tallying the number of studies by product type, method used and level of concern. Likert scale surveys and willingness-to-pay studies dominate this research (73.6%). Consumers tended to demonstrate concern regardless of product type. The only exception was beef, a product in which consumer concern was mixed.

While the majority of studies (106 studies) found some measurable level of consumer concern about antibiotic use in food production, far fewer studies investigated why consumers are concerned. Among all studies, 29.6% (37 studies) investigated why consumers are concerned about antibiotics. Among these, personal health and safety comprise half of the reasons given (67.6% including the safety category and all categories with “human health”; see table 4). The next most commonly cited reason for concern was animal welfare, comprising 32.4% of studies where perspectives were evaluated. It is notable that the evolutionary consequences of antibiotic

use—the emergence of antibiotic resistant bacteria in the world—is mentioned in only four studies (10.8% of those that examined reasoning) and this concern was always in combination with others. However, it is possible that concerns about antibiotic resistance were an unmentioned or implied aspect of human health and safety concerns.

The question regarding the demographics of individuals who share concerns about antibiotics in food production is also relatively neglected in the literature, only 24% (30 studies) of all studies. The most common descriptors across studies are gender (n=13), income (n=10), age (n=9), and education (n=6). In general, female, older, highly educated, and high-income were the demographic characteristics most consistently associated with consumer concern about antibiotics (see Table 5). While the findings for each of these features were consistent, there was at least one contradictory finding for each of these characteristics (e.g., one study found that men are more concerned about antibiotic use while all the others found more concern among women participants). Other personal identifiers included eating and shopping habits, level of trust, type of work, political views, ethical views, religion, race, awareness of the issue, location, and family structure. The results from these categories were found in few studies and without consistency across studies.

Although there are exceptions, questions aimed to investigate the politics of consumer choices and antibiotic use were ignored almost universally. Wolfe et al. (2016) conducted a large survey of consumers which found that two-thirds would vote hypothetically to restrict antibiotic use to medical treatment only, and men were more likely to reject such a policy. Conversely, individuals with higher incomes and those exposed to animal welfare media were more likely to

vote for such a policy. Goddard et al. (2019) examined the link between people's moral foundations and their attitudes toward purchasing and voting decisions for various credence attributes. They found that those who agreed with individualizing moral foundation statements (ethical concerns centered around impacts on individuals rather than commitment to the concerns of a wider social group) were more likely to purchase antibiotic-free products and also more likely to vote to ban such products compared to those who did not agree with such moral foundation statements. Finally, Lusk et al. (2007) conducted a willingness-to-pay study that showed consumers were both willing to pay more for antibiotic-free pork and also pay a premium for a ban on such products.

DISCUSSION

Research that investigates consumer concern about antibiotic use in animal agriculture production is gaining traction. Two-thirds of studies that met our inclusion criteria were published within the past ten years. This trend may relate to an increased public awareness and popularization of antibiotic-free and organic products, but longitudinal analysis was not conducted to confirm this theory.

Overall, consumer perceptions of antibiotic use in animal agriculture is overwhelmingly negative. Out of the studies that measured a degree of consumer concern (n=106), 77.4% found that there is some level of concern. This is unsurprising, given the number of studies that show consumer concern about potential practices that can be conceived as "contamination" (Brewer and Rojas 2008). While we have not completed reviews outside the ambit of antibiotics, several

studies found that genetically modified foods (Wunderlich and Gatto 2015), pesticides, (Boccaletti et al. 2000), and hormones (Lusk et al. 2003) are also of great concern to consumers.

Most studies indirectly measured antibiotic concern through credence labels (e.g., "raised without antibiotics" and "USDA Organic"), rearing practices, and food safety research in which antibiotic use is one of several related practices that were studied. Thus, in many cases, we had to extract the antibiotic-related findings from a study that was exploring a wider issue. This demonstrates a large gap in the literature, few studies were designed to assess consumer perspectives on antibiotic use as their primary focus.

WHY ARE CONSUMERS CONCERNED ABOUT ANTIBIOTICS IN ANIMAL AGRICULTURE?

While the reviewed papers demonstrate that consumers tend to be concerned about antibiotic use in animal agriculture, there are mixed findings as to why consumers are concerned. Although few studies (24%) investigated why consumers are concerned, findings indicate interesting and inconsistent trends. Primarily, consumers are concerned about health and safety, and then animal welfare.

Consumers who expressed reasons for concern may be ill-informed about animal agriculture production processes and antimicrobial uses. For example, consumers cited concerns that administration of antimicrobials in animals may present health and safety hazards to consumers. Although without further investigation, we cannot say what exactly those concerns are, one

conjecture is that consumers believe that drug administration leads to antibiotic residues on or in animal products that could contribute to consumer exposure to active antimicrobial agents (National Chicken Council 2015). However, the United States has strict regulations about antibiotic residues in animal products (FDA 2018). For example, the U.S. Department of Agriculture (USDA), in concert with the FDA and Environmental Protection Agency, founded the U.S. National Residue Program, monitors residues in meat through its Compound Evaluation System. This ensures the risk of exposure to antimicrobial residues in meat is low (NRC 1999). Similar regulatory efforts exist for non-meat animal products. It is possible that consumer's concern for human health is, in fact, expressing an unstated concern around antimicrobial resistance, however, none of the papers explored this potential conflation of these two terms. From a producer perspective, consumer concerns about animal welfare may appear similarly misguided. Some have argued that reducing on-farm antibiotic use is often worse for animal welfare because of the increased number of infections that tend to accompany this move (Karavolis et al. 2018, Singer et al. 20190).

Consumers may not, and likely do not, understand the nuances of antimicrobial use in animal production, specifically in terms of disease treatment, metaphylaxis, prophylaxis, and growth promotion/feed efficiency. Consumer knowledge about these complexities is hard to evaluate, and no studies addressed the terms with depth. Primarily, consumers associated antibiotic use with intensive animal production, lower animal welfare, and poor animal health. The reality from the producer side, however, is more nuanced, as animals may become infected with bacteria or other infectious agents even under optimized husbandry conditions, and according to producers, maintaining good animal welfare means treating animals when they are sick (Singer et al. 2019).

This producer-centric view is more attuned to the complex trade-offs involved in using antibiotics, but is also indicative of a sizable gulf in the attitudes between consumers and producers with regards to the relationship between antibiotic use and animal welfare. Singer et al.'s (2019) survey of producers shows that they are aware of this gulf of understanding, even if consumers are not. They found that producers felt that consumers believe raising animals without antibiotics would have significant improvement on animal husbandry, even as producers themselves did not believe this.

Abrams et al.'s (2010) qualitative study of pork consumers suggests that labels are a fairly effective and often used signaling device for consumers who wish to avoid potential risks related to health and safety. While experts in animal production can point to statistics on the low prevalence of antibiotic residue found on meat, this work suggests that lay consumers tend to latch on to an easily understood, qualitative marker of risk. In the case of pork meat, the prevalence of labels provides a quick and understandable signal of risk reduction. The prevalence of labels stating the absence of antibiotics (the "no" labels as the authors refer to them) provide a convenient guide for consumers who are making many of their food purchasing decisions in a compressed time period.

Typically, when discordance is found between consumer perceptions and producer realities, it is often accompanied by a call to "better educate" the consumer. We reject that "better education" will lead to different results. Instead, we posit that consumers are not ignorant or irrational, but, in fact, operate from a fairly rational and well-educated position. A closer look at the qualitative investigations into why consumers are concerned shows a fairly knowledgeable base of

consumers in terms of how the food system works. What is common across these studies, however, is that some consumers have associated antibiotic use as part and parcel of a demonized view of the industrialized food system. Sonntag et al. (2019), for example, found a wide range of consumer knowledge—from accurate understanding to misinformation—but a fairly consistent attachment between antibiotic use and an industrial process that is regarded as unhealthy for chicken and, by extension, people.

“Better education” is not necessarily an inappropriate intervention, however, available evidence in this review suggests it may not be effective as the only device that bridges the knowledge gap between producers and consumers, especially given that consumer antibiotic use concerns are tied to their negative feelings about modern industrial production systems. The relative paucity of research into why consumers are concerned about antibiotics shows that there is clearly more work to be done in this area. The literature to date has largely focused on how much consumers are willing to pay, or on quantifying the level of consumer concern. Unfortunately, the literature has not yet addressed the emotive attachments consumers have to food, the kinds of decision-making processes they make while in the grocery store, and the sorts of values beyond price they have when making purchasing decisions. Researchers may do well to consider ethnographic or other qualitative techniques to elucidate these questions.

WHO ARE THE CONCERNED CONSUMERS?

The literature has not comprehensively characterized individuals who may or may not be concerned about antibiotic use in animal agriculture. There were 24 studies that addressed this

question, and of these studies 14 different variables were identified as significant indicators of consumer concern. The most common variables found to be significant were gender, age, education, and income. Collectively, these studies illustrate that older, highly educated, high income females are most concerned about antibiotics. This picture of the “concerned consumer” is not a surprising one, and indeed, seems to play into the stereotype that organic food often serves as a luxury item for upper-class consumers. Nevertheless, these findings were not consistent across studies, and other, less explored variables were implicated in these papers that paint a potentially more complex picture of the concerned consumer.

There were a host of other characteristics found to be of significance, but they were limited to just a few studies, with little consistency in findings. Both “high trust” and “low trust” individuals were found to be concerned along with “altruistic people” and those with “individualizing moral foundations.” Both “Protestants” and “atheists” were also found to be concerned. These differences could be the result of different methods and/or the differences in study populations that researchers utilized. Perhaps with more research more stable typologies will emerge as we have seen with gender, income, education, and age.

One small (three studies) but consistent finding is that a consumer with a high level of knowledge and awareness tends to be concerned about antibiotics. Those with more knowledge seem to be more concerned, but as we discussed above, the kind of knowledge one has could greatly impact their stance on antibiotic use in animal industries. A high-knowledge consumer does not necessarily know specific information about antibiotic regimes and their role in animal production. Instead, “knowledge” often means a consumer understands the rules of thumb that

labels provide, or has a general understanding of how our food system works. We suggest here that the relationship between "high knowledge" consumers and concern about antibiotics further strengthens our contention to be wary of calls for further education of consumers. Such education is already being provided through labels, but it does not necessarily translate into a nuanced understanding of the role of antibiotic use in agriculture. Consumers have different ways of evaluating agricultural production than producers, and the evidence so far suggests that is unlikely to change.

Finally, the relative dearth of explicitly political studies (three studies) is surprising and indicates a clear need for further research. The so-called "vote/buy gap," where consumers will purchase a product that they will also vote to ban, is well documented in other literature (eg. Norwood et al. 2019). This gap points to the ways in which people compartmentalize their beliefs and actions. The opposite side of the vote/buy gap is the growing visibility of consumption choices as a form of politics (eg. Jackson et al. 2009). This can include campaigns to boycott particular products because of their owner's political views (Tomhave and Vopat 2018), or efforts to purchase products that meet ethical standards of production and trade (Johnston and Szabo 2011; Rossel and Schenk 2018). None of these political aspects of food consumption are covered by research into antibiotics and consumer preferences. Numerous economics studies have established the degree to which consumers will, or will not, pay extra money for antibiotic-free products. But with a few exceptions, none of these studies examine the extent to which these price preferences are related to political preferences with regard to agricultural policy. This is of particular concern because, as Paul et al. (2019) note, a potential gap between the public's consumption and voting behavior can complicate supply chain decision-making due to "increased uncertainty regarding

what ‘social license’ (e.g., freedom to operate) producers will maintain and what production practices will be accepted in the future” (pg. 102).

STUDY LIMITATIONS:

There are several limitations to this review. First, this review should not be considered generalizable to populations outside of the United States, Canada, the United Kingdom, and members-states of the European Union. Secondly, we only included manuscripts written in English. This may have biased findings, given that Canada and the European Union have multiple official languages, and this review may have excluded relevant literature that was written in non-English languages. Similarly, selection bias may have occurred because we required that studies have primary data collection with transparent and extractable methods and results. Many excluded works were grey literature sources produced by industry members. Thus, this research is skewed to peer-reviewed literature conducted by academic institutions.

CONCLUSION

This review was prompted by our interest in consumer perceptions about antimicrobial use in animal agriculture. Initial readings about this topic indicated that reasons for consumer concern are wide-ranging and consumer confusion exists about the use of antimicrobials in animal agriculture. Despite confusion, consumer perceptions are an important influence on animal agriculture practices. To understand what consumers see as the risks and benefits of antimicrobial use in animal agriculture, and to gauge which research and methodological gaps exist in this literature, we conducted a scoping review. Through an exhaustive search strategy

and systematic screening process, we identified 125 texts that fulfilled our inclusion criteria. We extracted relevant data from these texts for analysis, including the available data on consumer concern. The majority of studies used quantitative methods, willingness-to-pay studies and Likert surveys prominent among them, and were conducted by university researchers on U.S. populations. The studied products and themes varied.

Not every text measured consumer concern, and fewer assessed reasons for concern or identified characteristics of concerned people. Those that measured concern focused on antibiotic use, a priority to reduce antimicrobial resistance. The different topics of interest and methods used made synthesis of findings about consumer concern difficult. We developed a rubric to categorize each study's population into “concern,” “mixed concern,” or “no concern” regarding antibiotic use in animal agriculture. Most studies found some level of concern or mixed concern. Concern for human and animal welfare were the most common reasons cited. The animal welfare concern may derive from the consistent associations that consumers construe between antimicrobial use and industrial agriculture practices that they perceive as having negative consequences for the produced animals. It is notable that the emergence of resistant bacteria, which is a consequence of antibiotic use, is only mentioned in four studies and never as a study’s explicit focus.

Consumers may not understand the nuances of antimicrobial use in animal agriculture or specifics about disease treatment, metaphylaxis, prophylaxis, and growth promotion/feed efficiency uses. However, we do not recommend the typical tactic to educate consumers given that consumers may already be well informed about some aspects of animal production. We do

propose that more research should focus on consumer concern about antimicrobial use rather than appending a few questions about antimicrobial use to a study that has a broader focus. Similarly, more in-depth qualitative research is also needed on this topic because the overwhelming use of quantitative methods does not allow for a more nuanced understanding of consumer decisions. Further research into the politics surrounding consumer beliefs and decisions could be especially valuable as other research has evidenced a vote/buy gap between what people claim to be of importance and their purchasing decisions.

The dominance of university researchers and U.S. studies likely resulted from inclusion criteria that required texts be in English and have primary data collection. We cannot say if a more expansive criteria would lead to others results, but there were several seemingly relevant studies that could be incorporated into a future review. We also recognize that our criteria was limiting in the sense that non-academic types of literature (e.g. opinion pieces) were, with few exceptions, not captured and/or excluded. Future research into these other types of literature could be beneficial to further explain consumer perceptions and identify how these perceptions are acquired.

Acknowledgements:

The authors sincerely thank Alison E. Stout and Genevieve Jones for their efforts in searching for grey literature and screening captured texts. This manuscript is the result of collaborations formed during the Human Dimensions of Antimicrobial Resistance in Agriculture Workshop in Nebraska City, NE, USA in May 2019. This research was funded by USDA-NIFA grant number

2019-67017-29114, which allowed for two in-person meeting of the co-authors, and USDA-NIFA grant number 2018-68003-27467, which provided research support for JRB and DL.

REFERENCES

Arksey, H., & O'Malley, L. (2005). Scoping studies: towards a methodological framework. *International Journal of Social Research Methodology*, *8*(1), 19–32. https://doi.org/10.1080/1364557032000119616

Bishop, P. A., & Herron, R. L. (2015). Use and Misuse of the Likert Item Responses and Other Ordinal Measures.*International Journal of Exercise Science*, *8*(3), 297–302.

Boccaletti, S., & Nardella, M. (2000). Consumer willingness to pay for pesticide-free fresh fruit and vegetables in Italy. *The International Food and Agribusiness Management Review*, *3*(3), 297–310. https://doi.org/10.1016/S1096-7508(01)00049-0

Brewer, M. S., & Rojas, M. (2008). Consumer Attitudes Toward Issues in Food Safety. *Journal of Food Safety*, *28*(1), 1–22. https://doi.org/10.1111/j.1745-4565.2007.00091.x

California Legislative Body. Senate Bill No. 27 Livestock: use of antimicrobial drugs. . California Legislative Counsel; 2015. Available from: https://leginfo.legislature.ca.gov/faces/billTextClient.xhtml?bill_id=201520160SB27

CFR - Code of Federal Regulations Title 21. (n.d.). Retrieved March 31, 2021, from https://www.accessdata.fda.gov/scripts/cdrh/cfdocs/cfcfr/CFRSearch.cfm?CFRPart=556

Davis, M. F., & Rutkow, L. (2012). Regulatory Strategies To Combat Antimicrobial Resistance of Animal Origin: Recommendations for a Science-Based U.S. Approach. *Tulane Environmental Law Journal*, *25*(2), Article 2. https://journals.tulane.edu/elj/article/view/2291

Foundation I.F.I.C. (2018). 2018 Food and Health Survey. Retrieved on August 2, 2019 from https://foodinsight.org/2018-foodand-health-survey

Goddard, E., Hartmann, M., & Klink-Lehmann, J. (2017). Public Acceptance of Antibiotic Use in Livestock Production Canada and Germany. *Proceedings in Food System Dynamics*, *0*(0), 424–437. https://doi.org/10.18461/pfsd.2017.1743

Innes GK, Randad PR, Korinek A, Davis MF, Price LB, So AD, et al. External Societal Costs of Antimicrobial Resistance in Humans Attributable to Antimicrobial Use in Livestock. Annu Rev Public Health. 2020;No. 26189. https://doi.org/10.1146/annurev-publhealth-040218-043954

Jackson, P., Ward, N., & Russell, P. (2009). Moral economies of food and geographies of responsibility. *Transactions of the Institute of British Geographers*, *34*(1), 12-24.

Johnston, J., & Szabo, M. (2011). Reflexivity and the Whole Foods Market consumer: The lived experience of shopping for change. *Agriculture and Human Values*, *28*(3), 303–319. https://doi.org/10.1007/s10460-010-9283-9

Karavolias, J., Salois, M. J., Baker, K. T., & Watkins, K. (2018). Raised without antibiotics: Impact on animal welfare and implications for food policy. *Translational Animal Science*, *2*(4), 337–348. https://doi.org/10.1093/tas/txy016

Kirchhelle, C. (2018). Pharming animals: A global history of antibiotics in food production (1935–2017). *Palgrave Communications*, *4*(1), 1–13. https://doi.org/10.1057/s41599-018-0152-2

Landers, T. F., Cohen, B., Wittum, T. E., & Larson, E. L. (2012). A Review of Antibiotic Use in Food Animals: Perspective, Policy, and Potential. *Public Health Reports*, *127*(1), 4–22.

Lusk, J. L., Roosen, J., & Fox, J. A. (2003). Demand for Beef from Cattle Administered Growth Hormones or Fed Genetically Modified Corn: A Comparison of Consumers in France, Germany, the United Kingdom, and the United States. *American Journal of Agricultural Economics*, *85*(1), 16–29.

McDonalds-Global-Vision-for-Antimicrobial-Stewardship-in-Food.pdf. (n.d.). Retrieved August 2, 2019, from https://corporate.mcdonalds.com/content/dam/gwscorp/scale-for-good/McDonalds-Global-Vision-for-Antimicrobial-Stewardship-in-Food.pdf

Medicine, C. for V. (2021a). FACT SHEET: Veterinary Feed Directive Final Rule and Next Steps. *FDA*. https://www.fda.gov/animal-veterinary/development-approval-process/fact-sheet-veterinary-feed-directive-final-rule-and-next-steps

Medicine, C. for V. (2021b). Veterinary Feed Directive (VFD). *FDA*. https://www.fda.gov/animal-veterinary/development-approval-process/veterinary-feed-directive-vfd

National Chicken Council . 2015. Chicken check in 2015. Accessed March 8, 2019. https://www.chickencheck.in/media/nationwide-survey-reveals-nearly-80-percent-of-americans-mistakenly-believe-that-chicken-contains-hormones-or-steroids/.

National Research Council (US) Committee on Drug Use in Food Animals. (1999). *The Use of Drugs in Food Animals: Benefits and Risks*. National Academies Press (US). http://www.ncbi.nlm.nih.gov/books/NBK232571/

Norwood, Franklin Bailey, Glynn Tonsor, and Jayson L. Lusk. "I will give you my vote but not my money: Preferences for public versus private action in addressing social issues." *Applied Economic Perspectives and Policy* 41.1 (2019): 96-132.

Oliver, S. P., Murinda, S. E., & Jayarao, B. M. (2011). Impact of antibiotic use in adult dairy cows on antimicrobial resistance of veterinary and human pathogens: A comprehensive review. *Foodborne Pathogens and Disease*, *8*(3), 337–355. https://doi.org/10.1089/fpd.2010.0730

Paul, A. S., Lusk, J. L., Norwood, F. B., & Tonsor, G. T. (2019). An experiment on the vote-buy gap with application to cage-free eggs. *Journal of Behavioral and Experimental Economics*, *79*, 102–109. https://doi.org/10.1016/j.socec.2019.02.005

Pinsky, Benson, Conway, Currie, Feldman, Ferguson, et al. Senate Bill 422: Keep Antibiotics Effective Act of 2017. 2017 p. 1–5.

Polansek, T. (2014, December 10). Big U.S. school districts plan switch to antibiotic-free chicken. *Reuters*.https://www.reuters.com/article/us-antibiotics-chicken-education-idUSKBN0JO00320141210

Robinson, T. P., Bu, D. P., Carrique-Mas, J., Fèvre, E. M., Gilbert, M., Grace, D., Hay, S. I., Jiwakanon, J., Kakkar, M., Kariuki, S., Laxminarayan, R., Lubroth, J., Magnusson, U., Thi Ngoc, P., Van Boeckel, T. P., & Woolhouse, M. E. J. (2016). Antibiotic resistance is the

quintessential One Health issue. *Transactions of the Royal Society of Tropical Medicine and Hygiene*, *110*(7), 377–380. https://doi.org/10.1093/trstmh/trw048

Rössel, J., & Schenk, P. (2017). How Political is Political Consumption? The Case of Activism for the Global South and Fair Trade. *Social Problems*, spx022. https://doi.org/10.1093/socpro/spx022

Singer, R. S., Porter, L. J., Thomson, D. U., Gage, M., Beaudoin, A., & Wishnie, J. K. (2019). Raising Animals Without Antibiotics: U.S. Producer and Veterinarian Experiences and Opinions. *Frontiers in Veterinary Science*, *6*. https://doi.org/10.3389/fvets.2019.00452

Tomhave, A., & Vopat, M. (2018). The business of boycotting: Having your chicken and eating it too. *Journal of Business Ethics*, *152*(1), 123-132.

Tricco, A. C., Lillie, E., Zarin, W., O'Brien, K. K., Colquhoun, H., Levac, D., Moher, D., Peters, M. D. J., Horsley, T., Weeks, L., Hempel, S., Akl, E. A., Chang, C., McGowan, J., Stewart, L., Hartling, L., Aldcroft, A., Wilson, M. G., Garritty, C., … Straus, S. E. (2018). Prisma extension for scoping reviews (Prisma-scr): Checklist and explanation. Annals of Internal Medicine, 169(7), 467–473. https://doi.org/10.7326/M18-0850

Veterinary Feed Directive. (2015, June 3). Federal Register. https://www.federalregister.gov/documents/2015/06/03/2015-13393/veterinary-feed-directive

Wunderlich, S., & Gatto, K. A. (2015). Consumer Perception of Genetically Modified Organisms and Sources of Information. *Advances in Nutrition*, *6*(6), 842–851. https://doi.org/10.3945/an.115.008870

Tables:

Table 1: Timeline and Source Characteristics from the Extracted Texts

**Study Characteristics**	**No.**	**%**
Publication Date		
Pre-2009	38	30.4%
2010-2015	34	27.2%
2016-2020	53	42.4%
Publication Type		
Academic Journal	84	67.2%
Book	1	0.8%
Dissertation	7	5.6%
Thesis	5	4.0%
News Article	9	7.2%
White Paper	2	1.6%
Report	7	5.6%
Trade Journal	4	3.2%
Conference/Workshop Paper	3	2.4%
Website	1	0.8%
Datasheet	2	1.6%
Author Affiliation		
University	90	72.0%
Government	7	5.6%
Experiment Station	2	1.6%
Industry	8	6.4%
Think Tank	2	1.6%
Advocacy Group	1	0.8%
University and Government	1	0.8%
University and Industry	1	0.8%
Government and Industry	2	1.6%
Group/Association	4	3.2%
Unspecified	7	6.4%
Country of Study		
United States	68	54.4%
Canada	12	9.6%
Germany	7	6.4%
Single European Union Country	17	13.6%
United States and Canada	3	2.7%
Multiple European Union Countries	10	8.0%
Mixed European and North American Countries	5	4.0%
Unspecified	3	2.4%

Table 2: Product and Theme Focus of Extracted Texts

Study Characteristics	No.	%
Product		
Beef	17	13.6%
Pork	19	15.2%
Poultry	13	10.4%
Dairy	13	10.4%
Seafood	6	4.8%
Other Single Products	1	0.8%
Mixed Products	19	15.2%
Generic Categories	30	24.0%
Unspecified	7	5.6%
Themes		
Antibiotic Use	16	12.8%
Production Characteristics	29	23.2%
Food Safety	20	16.0%
Credence Attributes	13	10.4%
Organic	8	6.4%
Labels	8	6.4%
Food Quality	6	4.8%
Animal Welfare	6	4.8%
Risk	5	4.0%
Natural	3	2.4%
Environmental Concerns	2	1.6%
Trust	2	1.6%
Purchasing/Marketing	2	1.6%
Parent Decisions	1	0.8%
Performance Enhancers	1	0.8%
Regulation	1	0.8%
Social Welfare	1	0.8%
Vaccinations	1	0.8%

Table 3: Methods Used by the Extracted Texts

**Study Characteristics**	**No.**	**%**
Study Type		
Qualitative	14	11.2%
Quantitative	103	82.4%
Mixed Qualitative and Quantitative	8	6.4%
Data Collection Method		
Survey	70	56.0%
Choice Experiment	9	7.2%
Qualitative Method	8	6.4%
Document/Literature Analysis	8	6.4%
Mixed Methods	26	20.8%
Unspecified	4	3.2%
Likert or WTP Study		
Willingness-to-pay Study	43	34.4%
Likert Scale Study	49	39.2%

Table 4: Characteristics of Studies that Measured Level of Concern and Reasons for Concern

**Consumer Concern Indicators**	**No.**	**%**
Level of consumer concern for all 110 texts		
Concerned	82	65.6%
Mixed Concern	14	11.2%
Not Concerned	10	8.0%
Study Did Not Measure Concern	19	15.2%
Reason for consumer concern from the 37 studies included in this analysis		
Safety	9	24.3%
Human Health and Residues	10	27.0%
Human Health and Resistance	3	8.1%
Animal Welfare and Human Health	1	2.7%
Animal Welfare, Human Health and Antimicrobial Resistance	2	5.4%
Animal Welfare	8	21.6%
Animal Welfare and Resistance	1	2.7%
Production Practices	2	5.4%
Social Responsibility	1	2.7%

Table 5: Summary of findings from studies that gauged the types of consumers concerned about antibiotic use.

Type of Characteristic	No.	Specific concern variables	"Not concerned" variables	Example Paper
Gender	13	female (10); males; both (situation dependent)	Males(2)	Widmar 2017
Age	9	over 65, over 70, older (4), younger, old/young (situation dependent)	young	Yuxiang 2019
Income	10	higher income (8), lower income	higher income	Wolf et al. 2016
Education	6	university degree, more educated (3)	more educated(2)	Steiner and Yang 2010
Eating and Shopping habits	4	meat eaters, pork buying habits, shops at farmer's markets, household shopper	none	Bergstra et al. 2017
Level of trust	3	high trust, low trust (2)	none	Muringai 2016
Knowledge and awareness	3	label readers, "health mavens", production knowledge	none	Smith et al. 2017
Work	3	"housewives", union members, employed	none	Connor et al. 2008
Political views	3	socially aware, conservatives, social liberals	none	Ubilave et al. 2010
Ethical views	3	altruistic people, Individualizing moral foundation, believe that "organic" is better for cows	none	Lusk et al. 2007
Religion	3	Protestants, Atheists, religiousity	none	Bergstra et al. 2017
Race	3	non-white, Black, white	none	Steiner and Yang 2010
Location	2	Montana, Quebec	none	Veeman and Lee 2007
Family structure	1	parents with children under 6	none	Tong 2011

"N" is the total number of times the variable category was found to be significant across all papers. In sum, 52 variables across 30 different studies were found.

Figure Captions

Figure 1: Scoping Methodology (attached)

Figure 2: Tally of studies by food studied, method used, and level of concern about antibiotics that the study found. Excludes studies that did not explicitly gauge a level of concern about antibiotics and studies that did not specify the product. Each dot is one study.

Figure 1

Click here to access/download;Figure;LANSING PRISMA DIAGRAM UPDATED (1).doc

PRISMA 2009 Flow Diagram

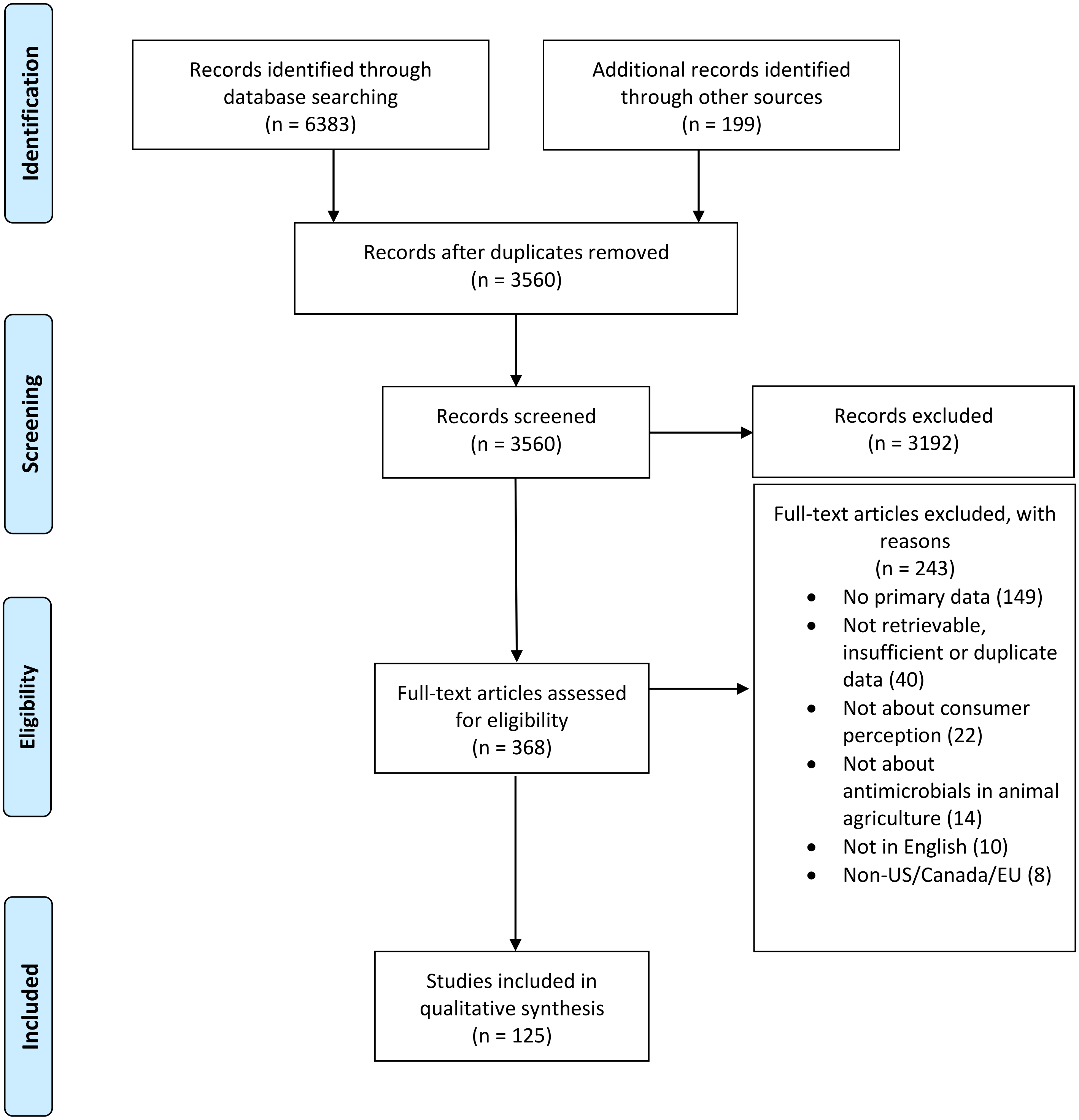

From: Moher D, Liberati A, Tetzlaff J, Altman DG, The PRISMA Group (2009). *P*referred *R*eporting *I*tems for *S*ystematic Reviews and *M*eta-*A*nalyses: The PRISMA Statement. PLoS Med 6(7): e1000097. doi:10.1371/journal.pmed1000097

For more information, visit www.prisma-statement.org.

	WTP	Likert	Qualitative	Total
**Beef**	⌀<br>●●●●●<br>◉	●●●<br>◉		⌀ 1<br>● 8<br>◉ 2
**Pork**	●●●●●<br>●<br>◉	●●●●●<br>●●●<br>◉	●	● 15<br>◉ 2
**Poultry**	●●●	⌀<br>●●●	●●<br>◉	⌀ 1<br>● 8<br>◉ 1
**Dairy**	⌀<br>●●●●	⌀<br>●●●<br>◉		⌀ 2<br>● 7<br>◉1
**Seafood**	●●	●		● 3
**Multiple Products**	●●●<br>◉◉	⌀<br>●●●●●<br>◉◉	◉	⌀ 1<br>● 8<br>◉ 5
**Generic “meat”**	⌀<br>●●●●●<br>●●	⌀⌀⌀<br>●●●●●<br>●●●●<br>◉	●●	⌀ 4<br>● 18<br>◉ 1
**Total**	⌀ 3<br>● 30<br>◉ 4	⌀ 6<br>● 32<br>◉ 6	● 5<br>◉ 2	⌀ 9<br>● 67<br>◉ 12

⌀ = study found no concern
● = study found concern
◉ = study found mixed concern

Supporting Document 1: Bibliography of final texts

Click here to access/download
Supporting Information
Supporting doc 1 Bibliography of Final Texts_Update.docx

Click here to access/download
Supporting Information
Supporting Document 2 Scoping Checklist_Update.docx
