# Supplementary material for: Consumer perceptions of antimicrobial use in animal husbandry: A scoping review
Source: PLoS One. 2021 Dec 8;16(12):e0261010. doi: 10.1371/journal.pone.0261010 (PMC8654221; doi:10.1371/journal.pone.0261010)
Supplement: Attachment — Submitted filename: Response to Reviewers.docx [file pone.0261010.s004.docx]

**Response to Reviewers**

| **Comment**  **No.** | **Reviewer No.** | **Comment** | **How we addressed** |
| --- | --- | --- | --- |
| 1 | Editor | Change title from "Animal agriculture" to "Animal husbandry" | Changed "agriculture" to "husbandry". Also dropped the geography from the title to avoid confusion of paper expectations. |
| 2 | 1 | the authors need to update the manuscript contribution | We are somewhat confused by what the reviewer is asking here. Based on our interpretation of the comment, we revised the title page to match the PLoS ONE guidelines for affiliation formatting. Additionally, we moved authors contributions to the end of the manuscript. However, if that is not what the reviewer asked, we would be happy to make additional changes following the reviewer’s further clarification. |
| 3 | 1 | the authors can suggest new research lines based on the review | Added three paragraphs to Conclusion about this. See lines 591-632. |
| 4 | 1 | the review title suggests geographical focus. as a result the reader would expect a geographical classification description of main results and discussions in each target location | Thank you. This was miscommunication on our part. We removed reference to the geographical focus from the title and added an extra line to first paragraph of results section, clarifying our goals. See lines 258-260. |
| 5 | 3 | Briefly explain "PRISMA guidelines" for general readers | Added language to end of first paragraph of Methods section to clarify what PRISMA is for general readers. See lines 115-116. |
| 6 | 4 | It is mentioned that the scope of the study was US, Canada and the EU. Were there articles published in English in these countries of interest but that were focused on investigating antibiotics perceptions in other countries (outside the EU, Canada and the U.S.? If so, it may be relevant to note this. | Added language to the methods section to clarify that our geographic criteria are about where the consumer populations are, not where it was published. See line 174-175. |
| 7 | 4 | Comparisons between concern levels for antibiotics used between U.S., Canada and the EU could be interested to look into. Did the authors considered this? | We did do some exploratory analysis along these lines but did not find strong trends. We feel that a deeper analysis of geographic differences of consumers is closer to a metaanalysis and is outside the ambit of a scoping review. We added text to the methods section to clarify the purpose of our geographic restrictions. See lines 202-209. |
| 8 | 4 | An important finding highlighted by the authors is that “Antimicrobial resistance rarely registered as an explicit reason for concern.” While “human health” was a prominent reason for concern. I think that it is possible that consumers in the studies analyzed may have understood that “human health” included “antibiotics resistance”. This possibility should be considered and addressed. | Added text to the conclusion to acknowledge this possibility. See lines 639-642. |
| 9 | 4 | “The most common and consistent features of these consumers were gender, age, income, and education.” But no findings from the analyzed articles were reported utilizing these demographics categories. I recommend removing this statement if it will not be directly used in the abstract. | This might have been a misunderstanding. We do indeed report findings from the analyzed articles about these demographic categories (see sub-section “Who are the concerned consumers” in the Discussion; also table 5). 2. We use this statement in the abstract, and follow up fairly extensively in the body of the paper on these categories.  . |
| 10 | 4 | “Overall, studies tended to be dominated by either willingness-to-pay studies or likert scale questionnaires (73.6% of all studies). The popularity of these methods may have contributed to the relative lack of studies that characterized worried consumer demographics or reasons for their perspectives.” But wtp and likert studies are not mutually exclusive with asking consumers why they hold certain views. This statement may misrepresent the extent of information that can be obtained from such study methodologies. | We removed this sentence (the one beginning “The popularity…”) from the abstract. |
| 11 | 4 | “We recommend more qualitative research into consumer views on this topic, which may better elucidate consumer decision-making and mentality” But it is not clear how asking qualitative questions to consumers will help with their decision making or mentality. It seems to me that educational campaigns may serve this purpose better then more qualitative research. Perhaps refrace? | Yes, this was a miscommunication on our part. We are not asking consumers to do anything, but merely reporting what other studies have done. We have rephrased this statement to avoid confusion on this point. See lines 57-59. |
| 12 | 4 | 122-124. “may influence” or “have some influence” may be more appropriate than “influence” | Agreed, changed to "may influence". See line 97. |
| 13 | 4 | 237. “We made this decision because these countries have similar regulatory environments and close trade associations” Do you mean similar regulatory environments when it comes to the use of antibiotics in animal agriculture? If so, you can include that in the manuscript | Added more clarification to introduction and methods about our choices of geographic restriction, including the regulatory environment. See lines 202-209. |
| 14 | 4 | 360. “The only exception was beef, a product in which consumer concern was mixed.” Do the authors have any idea or potential hypothesis as to why may this be the case? | We feel that there is not enough information in the papers to make such a speculation. The difference is by just a few papers, and the studies are all quite different. It is possible with more studies this difference would disappear. In any case, we don’t wish to speculate in this review. |
| 15 | 4 | 364-371 “Among all studies, 29.6% (37 studies) investigated why consumers are concerned about antibiotics. Among these, personal health and safety comprise half of the reasons given (67.6% including the safety category and all categories with “human health”; see table 4). The next most commonly cited reason for concern was animal welfare, comprising 32.4% of studies where perspectives were evaluated. It is notable that the evolutionary consequences of antibiotic use—the emergence of antibiotic resistant bacteria in the world—is mentioned in only four studies (10.8% of those that examined reasoning) and this concern was always in combination with others.” Is it possible that consumers who participated in these studies referenced understood “personal health and safety” to encompass “evolutionary consequences of antibiotic use”? This is something that needs to be carefully addressed by understanding in detail the verbiage used in each study. This may change some of the main findings from this review. | We addressed this the same way as comment no. 8 by adding text to the conclusion to acknowledge the possibility that these categories may encompass concern about AMR even if it is not explicitly mentioned. See lines 638-642. |
| 16 | 4 | We reject that “better education” will lead to different results.” On what basis is this rejected? | We have revised this discussion considerably. We cited and discussed literature that concluded this model of education is of limited efficacy and insufficient. We also softened our language, removing words like "reject" in favor of a more nuanced discussion of the kinds of education that may or may not work. See also our response to comment #23. See lines 470-495. |
| 17 | 4 | “especially given that consumer antibiotic use concerns are tied to their negative feelings about modern industrial production systems” needs citation | Added two citations here (line 480). |
| 18 | 4 | Both “Protestants” and “atheists” were also found to be concerned” Is this compared to other religious groups or faith associations? | Clarified this in second paragraph of "who are concerned consumers" subsection. It was one study that measured "non religious"; protestants, and Catholics. See lines 509-511. |
| 19 | 4 | One small (three studies) but consistent finding is that a consumer with a high level of knowledge and awareness tends to be concerned about antibiotics” Is this “self-reported knowledge” if not, how was knowledge level measured? | We clarify that consumers were asked about very basic knowledge about antibiotics. See lines 520-521. |
| 20 | 4 | “Consumers have different ways of evaluating agricultural production than producers, and the evidence so far suggests that is unlikely to change.” Please list this evidence | This sentence is eliminated from the new version. |
| 21 | 5 | For example, your categorization of different points of concern based on Likert scale ratings (page 11 L252-259) may be akin to comparing apples to oranges as the scales may have different ranges. I would advise considering the extreme ends and grouping the anything between the extremes together to avoiding the arbitrariness of using the average as the cut-off. Further, Willingness to pay (WTP) cannot be used as a measure of concern as WTP is strongly driven by income (ability to pay). There may be segments of consumers who care about antibiotic use but simply cannot afford to pay and therefore will report WTPs of zero. Instead of concern, why don’t categorized that as valuation? | Added recognition of this issue in the study limitations section. Unfortunately, for the stated reasons we were not able to make the changes the reviewer requests. We cover this issue extensively in the “limitations” section. See lines 560-581. |
| 22 | 5 | Your conclusion that consumer perceptions of antibiotics use is overwhelmingly negative (L409) appear not to be supported by your results. Indeed, in the subsequent line you note that 77.4% found some level of concern. This according to your analysis is due to a misunderstanding of the what antibiotics use is (i.e. perceived contamination) (Brewer and Rojas 2008). | It is possible consumer perception is based on faulty knowledge, but it is negative nonetheless. In this case, we want to understand perceptions regardless of their accuracy. See lines 391-395. |
| 23 | 5 | It is also quite surprising that you appear to strongly oppose consumer education although your work severally indicates that consumers are misinformed about antibiotics, their effect and the intersection with animal welfare, human health and AMR. I see no basis in your work to reject the notion of increased consumer education about the issue. | Thank you for this comment. We have revised our language on this in two ways. First, we have softened our claims around this issue. We do not oppose education, but instead, we clarify that one particular model of education, that of transmitting more scientific knowledge to the consumer, is unlikely to suffice. This is due to quite of bit of evidence out there that this model of science communication is ineffective. Instead, we draw on the few qualitative studies on the topic of consumer perceptions of antibiotics to suggest that forms of education need to take into account consumer's values and emotional attachments to food if it is going to be effective. See lines 470-495. |
| 24 | 5 | The issue of antibiotics use in pork production is huge due to the nature of diseases and its impact on production. This is different from cattle, for example. At the very least, authors should discuss these issues. Yes, I agree your focus is very narrow, but a good background and context enriches your discussion. The current version of this manuscript is severally lacking a thorough discussion of the themes identified in the literature review. | We added text to the discussion to discuss some of the key differences seen across different forms of production and antibiotic use. Such a discussion is necessarily very truncated but helps acknowledge that there are indeed differences across production systems. See lines 447-459. |
| 25 | 5 | Page 6 and 7 Line 143 and 144: You indicate that your study focuses on Canada, US and EU without providing a justification for the focus on these countries/regions. You wait until page 11 L237. The choice of the countries and the justification are an important piece of your study and have to be discussed much earlier in the text. Apart for the similarities in the regulatory environment. Are there other farm/consumer driven reasons for the choice? | We added a line to last paragraph of Introduction to flag this. We further expand on this in the Methods section. One other reason for limiting our studies to these areas is they all have a similar level of development, and thus consumer access to food choices. See lines 104-107 and lines 202-209. |
| 26 | 5 | L515- Trust in what? Is it institutional trust? | It is trust in food safety. This is clarified in the text. See lines 507-508. |
| 27 | 5 | L523-High level of knowledge in what? Is it level of education or education specific to antibiotics? | We added text to this paragraph to clarify that it is specific knowledge about antibiotics. See lines 520-521. |
| 28 | 5 | Goddard (2019) is missing in the reference list | Added Goddard et al. 2019 to bibliography. It is reference #23. |
| 29 | 2 | References needed for lines 147-165 | These are our group derived definitions. We added "we define…" where appropriate to make it clear these are our own definitions for the search terms. |
